# Comprehensive Analysis of Fecal Microbiome and Metabolomics in Hepatic Fibrosis Rats Reveal Hepatoprotective Effects of Yinchen Wuling Powder From the Host-Microbial Metabolic Axis

**DOI:** 10.3389/fphar.2021.713197

**Published:** 2021-07-27

**Authors:** Yumeng Zhang, Min Zhao, Xue Jiang, Qiaoyu Qiao, Tingting Liu, Chunjie Zhao, Miao Wang

**Affiliations:** ^1^ School of Pharmacy, Shenyang Pharmaceutical University, Shenyang, China; ^2^ School of Life Science and Biopharmaceutics, Shenyang Pharmaceutical University, Shenyang, China

**Keywords:** Yinchen Wuling powder, hepatic fibrosis, 16S rRNA gene sequencing, fecal metabolomics, correlation analysis, butyrate

## Abstract

Hepatic fibrosis (HF) is a typical consequence in the development of multiple chronic liver diseases, which is intimately related to the composition and metabolic status of gut microbiota. A myriad of evidence has indicated that traditional Chinese medicine can treat HF by regulating gut microbiota. Yinchen Wuling powder (YCWLP) is a famous traditional Chinese medicine prescription, which has been used to relieve liver diseases for thousands of years. YCWLP has demonstrated protective function on HF, but its effect on the alterations of gut microbiota is still unclear, and its explicit therapeutic mechanism also needs to be further elucidated. In this study, 16S rRNA gene sequencing and fecal metabolomics analysis were combined to investigate the influence of YCWLP on gut microbiota in HF rats and the interactions between gut microbiota and host metabolism. The results showed that YCWLP treatment significantly improved the disorder of multiple organ indices, HF-related cytokines and plasma LPS induced by HF. Masson’s trichrome stainings also showed that YCWLP treatment could significantly alleviate the severity of HF in rats. Additionally, YCWLP could reverse the significant changes in the abundance of certain genera closely related to HF phenotype, including *Barnesiella* [*Ruminococcus*] and *Christensenella*. Meanwhile, YCWLP significantly increased the abundance of *Bifidobacterium*, *Coprococcus* and *Anaerostipes*, which are closely related to butyrate production. Metabolomics and Spearman’s correlation analysis showed that YCWLP could regulate the disorder of arginine biosynthesis, sphingolipid metabolism and alanine, aspartate and glutamate metabolism in HF rats, and these regulations were intimately related to *Barnesiella*, [*Ruminococcus*]*, Christensenella, Coprococcus* and *Anaerostipes*. By explaining the biological significance of the above results, we concluded that YCWLP might ameliorate HF by regulating the imbalance of gut microbiota, increasing the abundance of butyrate-producing bacteria to reduce ammonia production, promote ammonia degradation, and regulate pro-inflammatory cytokines and immune function.

## Introduction

Hepatic fibrosis (HF) is the pathological basis of the progress of diverse chronic liver diseases, which is characterized by excessive deposition of extracellular matrix (ECM). The development of HF into cirrhosis will seriously endanger human health (Williams, 2006). Nevertheless, HF is pathologically reversible, and early treatment can effectively prevent its further development (Pinzani and Rombouts, 2004). In the past few decades, innumerable treatment modalities targeting HF have been studied, but these methods have hardly achieved substantial results.

Liver and intestine originate from the same germ layer, so liver and intestinal microecology are intimately related not only in anatomical position, but also in function. The liver directly interacts with the gut through the bile secretion system and hepatic portal, which is called the “gut-liver axis” (Wahlstrom et al., 2016). Accumulating numbers of researches have shown that gut microbiota is significantly related to chronic liver disease progression (Li et al., 2020). The occurrence of HF is usually accompanied by microbiome dysbiosis and the impairment of the intestinal barrier. The excessive growth of small intestine bacteria leads to the accumulation of a large number of toxic substances such as endotoxin and ammonia. These pathogens and toxins enter the liver through the damaged intestine and portal vein, bind with toll-like receptors (TLRs), and then activate the liver immune cells, produce inflammatory cytokines, induce the liver innate immune response, and finally aggravate the progress of HF (Guo and Friedman, 2010; Seki and Schnabl, 2012). Additionally, gut microbiota also participates in the occurrence, development and treatment of liver diseases through host-microbe metabolic axes (Nicholson et al., 2012). In recent years, it has become a research hotspot to prevent and treat HF by ameliorating gut microbiota disorder (Dhiman et al., 2014). It is of great significance to investigate the mechanism of drug therapy of HF with gut microbiota as the target.

Yinchen Wuling powder (YCWLP) is a famous traditional Chinese medicine (TCM) prescription for the treatment of liver diseases recorded in *Synopsis of the Golden Chamber* written by Zhang Zhongjing, a famous physician in the Eastern Han Dynasty. It is composed of *artemisia Capillaris Herba*, *Polyporus Umbellatus*, *Alismatis Rhizoma*, *Atractylodes Macrocephalae Rhizoma stir-fried with wheat bran*, *Poria* and *Cinnamomi Ramulus*. In modern clinics, it is commonly used in the treatment of icteric hepatitis (Ma, 2020), hepatic fibrosis (Chen, 2012), non-alcoholic fatty liver disease (Liu and Zhao, 2011) and other diseases. In our previous study, we investigated the hepatoprotective effects and underlying mechanism of YCWLP on CCl_4_-induced HF (Zhang et al., 2021). After 8 weeks of intervention, YCWLP could significantly reduce the serum aspartate transaminase (AST), alanine transaminase (ALT), hyaluronidase (HA), laminin (LN), type Ⅳ collagen (CIV) and N-terminal propeptide of procollagen type III (PIIINP) of HF rats. The histopathological analysis also showed that YCWLP could protect hepatocytes and reduce the degree of HF. Additionally, the metabolomic analysis of plasma, urine and liver samples also indicated that the mechanisms underlying the effects of YCWLP might be through reducing ammonia accumulation, promoting energy metabolism, reducing oxidative stress, protecting cell membrane and regulating intestinal flora metabolism. We have noticed that the mechanism of YCWLP in the treatment of HF might be related to gut microbiota. The increasing number of evidence also suggests that TCMs may be used as prebiotics to regulate the composition of gut microbiota and the metabolic phenotype of the host, and further as a new source for drug leads in gut microbiota-targeted disease management (Xu et al., 2015; Chang et al., 2017; Yu et al., 2017). Therefore, this study further elucidated the mechanism of YCWLP in the treatment of HF from the perspective of gut microbiota.

Fecal metabolomics can better reflect the intestinal-related metabolites, its association with the functional readout of the intestinal microbiome is of great value for the understanding of the microbiota-metabolism interactions (Zierer et al., 2018). Therefore, in this study, 16S rRNA gene sequencing analysis was used to analyze the changes of gut microbiota in CCl_4_-induced HF rats and the intervention effect of YCWLP. Furthermore, the interaction between gut microbiota and host metabolism was explored by combining 16S rRNA gene sequencing with fecal metabolomics to elucidate the mechanism of YCWLP in the treatment of HF.

## Materials and Methods

### Drugs and Chemicals

Artemisia Capillaris Herba (batch number: 190301; source: Hebei, China), Polyporus Umbellatus (batch number: 1808079; source: Liaoning, China), Alismatis Rhizoma (batch number: 190705; source: Sichuan, China), Atractylodes Macrocephalae Rhizoma stir-fried with wheat bran (batch number: 200622; source: Anhui, China), Poria (batch number: 190601; source: Anhui, China) and Cinnamomi Ramulus (batch number: 190401; source: Guangxi, China) were purchased from Shenyang Guoyaoda Pharmacy (Shenyang, China) and authenticated by Prof. Jia from Shenyang Pharmaceutical University (Shenyang, China).

HPLC grade acetonitrile, HPLC grade methanol and LC/MS grade formic acid were purchased from Fisher Scientific (Fair Lawn, NJ, United States). Analytical grade dibasic sodium phosphate and sodium dihydrogen phosphate were obtained from Kemeo Regent Co., Ltd (Tianjin, China). Sodium 3-trimethylsilyl-propionate [2,2,3,3,d_4_] (TSP) and deuteroxide (D_2_O) were supplied by Merck Drugs & Biotechnology (Darmstadt, Germany). Purified water was bought from Wahaha Co., Ltd. (Hangzhou, China). Masson’s trichrome staining kit was purchased from Beijing Solarbio Science & Technology Co., Ltd. Platelet derived growth factor (PDGF), transforming growth factor-β1 (TGF-β1), tissue inhibitor of matrix metalloproteinases-1 (TIMP-1), α-smooth muscle actin (α-SMA) and lipopolysaccharide (LPS) assay ELISA kits were provided by Jiangsu Meimian Industrial Co., Ltd. (Jiangsu, China).

### Preparation of Yinchen Wuling Powder Suspension

All crude herbal medicines were ground into powder and then sieved through a 60 mesh stainless steel sieve. The dosage of each drug in YCWLP used in this study was in accordance with the record of “*Synopsis of the Golden Chamber*”. 6 g YCWLP contained: 4.000 g of Artemisia Capillaris Herba, 0.375 g of Polyporus Umbellatus, 0.625 g of Alismatis Rhizoma, 0.375 g of Atractylodes Macrocephalae Rhizoma stir-fried with wheat bran, 0.375 g of Poria and 0.250 g of Cinnamomi Ramulus. The above herbs were then mixed with 60 ml normal saline to obtain the YCWLP suspension (0.1 g/ml). Besides, the suspension was prepared before each administration and mixed evenly before each rat was given intragastric administration.

### Animal Experiments

18 male Sprague-Dawley (SD) rats (200 ± 20 g) supplied by the Experimental Animal Center of Shenyang Pharmaceutical University (Liaoning, China) were maintained in an environmentally controlled room (12/12 light-dark cycle, 20–25°C, 40%–70% relative humidity) with *ad libitum* access to food and water. All animal protocols in this study were approved by the Medical Ethics Committee of Shenyang Pharmaceutical University and in accordance with the guidelines of the National Institutes of Health on Animal Care (2004).

After 1 week of acclimation, all animals were randomly divided into three groups (*n* = 6): the control group (CON group), the hepatic fibrosis group (HF group) and the YCWLP treatment group (YCWLP group, 2 g/kg/d). The rats in the HF and YCWLP groups were orally administrated with 40% (v/v) CCl_4_ (dissolved in soybean oil, 2 ml/kg) twice a week for 13 weeks, whereas the CON rats obtained the same dose of soybean oil with the same procedure. Since the 6th week, the rats in the YCWLP group were administered with YCWLP suspension once daily for 8 weeks, while the rats in the CON and HF groups received an equivalent volume of normal saline.

### Sample Collections and Preparation

At the end of the 13th week, fecal samples within 12 h were collected on ice using sterilized metabolism cages under specific pathogen-free (SPF) conditions for metabolomics analysis. As for 16S rRNA gene sequencing analysis, fecal samples from each group were simultaneously obtained under sterile conditions in a laminar flow bench. The specific operation steps were as follows: disinfect the perianal area and tails of rats with 75% alcohol cotton, and collect fecal samples with aseptic freezing tubes using the tail-lifting method. All fecal samples above were stored at −80°C for further analysis immediately after collection.

Then, all rats were fasted overnight before the blood of the orbital venous plexus was collected and transferred to heparinized tubes. The plasma samples were obtained by centrifuging at 1,100 g for 15 min, and the supernatants were stored at −80°C for the determination of LPS. After all rats were sacrificed under ether anesthesia, the liver, spleen and thymus of each rat were removed and washed with pre-cooled normal saline. These organs were weighed after wiping dry and the organ indexes were calculated based on the percentages of organs to body weights. Finally, the liver of each rat was divided into two parts. One part was immediately snap-frozen in liquid nitrogen and stored at −80°C for the analysis of HF-related cytokines and the other part was fixed in 10% formaldehyde for Masson’s trichrome staining.

### Masson’s Trichrome Staining

The liver tissues were fixed in 10% formaldehyde for 24 h, embedded in paraffin and cut into 5 μm sections, then stained with Masson’s trichrome staining kit according to the manufacturer’s instructions. The liver histological and fibrotic changes were assessed by two pathologists who were blinded to the treatment protocol. The degree of HF was scored using the Ishak scoring system after observation under a biological microscope (Andruszkow et al., 2019).

### HF-Related Cytokines in Liver

After thawed at 4°C, 1.0 g liver tissue of each rat was immersed in 10.0 ml PBS (Na_2_HPO_4_−NaH_2_PO_4_, 0.01 M, pH = 7.4) followed by homogenized adequately to obtain liver tissue homogenate. Then, the homogenate was centrifugated at 1,100 g for 20 min and the supernatant was used to measure the activities of PDGF, TGF-β1, TIMP-1 and α-SMA by commercially available kits according to the manufacturer’s instructions. The optical density values of PDGF, TGF-β1, TIMP-1 and α-SMA were all measured at 450 nm by a microplate reader.

### Lipopolysaccharide

Plasma LPS was detected using a commercially available kit according to the manufacturer’s instructions, and the optical density value of each plasma sample was detected by a microplate reader at 450 nm.

### Fecal Metabolomics Analysis

#### ^1^H NMR Analysis

After thawed at 4°C, 200 mg feces of each sample was extracted with 1.0 ml pre-cooled PBS (Na_2_HPO_4_-NaH_2_PO_4_, 0.2 M, pH = 7.4) by ultrasound for 30 min. After centrifuged at 11,600 g for 10 min at 4°C, the supernatant was obtained. Finally, 450 μl supernatant was mixed with 100 μL TSP D_2_O solution (1.0 mg/ml) and transferred to a 5 mm NMR tube for ^1^H NMR analysis.

All fecal samples were analyzed at 298.2 K using Bruker AV 600 MHz superconducting Fourier transform NMR spectrometer (Bruker, Karlsruhe, Germany) with a one-dimensional water pre-saturated standard NOESYPR 1D pulse sequence. The pre-saturation method was used to suppress the water peak. Deuterium (D_2_O) + water (H_2_O) was used for field frequency locking and TSP was used as chemical shift reference (^1^H, 0.00ppm). ^1^H NMR spectra were measured with 64 scans into 64 K data points over a spectral width of 12,019 Hz and a relaxation delay of 2 s. An exponential function corresponding to a line broadening factor of 0.3 Hz was applied to all acquired free induction decays (FIDs) before Fourier transformation.

The obtained ^1^H NMR spectra were manually phased and baseline corrected using MestReNova 6.1.1 software (Mestrelab Research, United States) with TSP as the reference. The integral interval was δ 0–10.0 with an integral spacing of 0.04 ppm. Moreover, in order to eliminate the influence of water peak, the integral value of δ 4.7–5.2 was removed.

#### UPLC-MS Metabolomics Analysis

After thawed at 4°C, 250 mg of each fecal sample was mixed with 1.0 ml pre-cooled water, extracted by ultrasound for 20 min, and then centrifuged at 11,600 g for 10 min at 4°C to obtain the supernatant. 400 μl pre-cooled methanol and 400 μl pre-cooled acetonitrile that act as the extraction solvent were successively added to the precipitation, and the above steps were repeated after each addition of solvent. Finally, the supernatant obtained was mixed and centrifuged at 11,600 g for 10 min at 4°C, and filtered through a 0.22 μm microporous membrane for UPLC-MS analysis. Furthermore, 10 μl of each fecal supernatant was mixed to obtain a quality control (QC) sample.

UPLC analysis was conducted on the Waters ACQUITY UPLC system (Waters Corp., Milford, United States). Chromatographic peaks were separated on a universal XB C18 column (150 mm × 2.1 mm, 1.8 μm; Kromat, United States) at the temperature of 35°C. The mobile phase composed of 0.1% (v/v) formic acid-water (A) and 0.1% (v/v) formic acid-acetonitrile (B), the flow rate was 0.2 ml/min and the elution procedure was set as follows: 5–30% (B) in 0–8 min, 30–60% (B) in 8–10 min, 60–90% (B) in 10–15 min, 90–5% (B) in 15–15.1 min, 5% (B) in 15.1–20 min. The sample injection volume was set at 5 μl. MS analysis was carried out on the Waters Quattro mass spectrometer coupled with a triple quadrupole mass analyzer (Waters Corp., Milford, MA, United States) in positive ion mode. The MS conditions were as follows: the cone voltage was 35 V; the capillary voltage was 3.2 kV; the desolvation temperature was 350°C; the source temperature was 120°C; the desolvation flow rate was 600 L/h; the cone gas flow rate was 50 L/h; the collision energy was 10–30 eV. In addition, the mass range was set from 100 to 1,000 Da with full scan mode.

The repeatability and stability of the UPLC-MS platform were evaluated using QC samples by the method described in our previous study (Zhang et al., 2021). Finally, the original data were imported into Markerlynx V4.1 (Waters Corp., Milford, United States) for peak deconvolution and normalization and a matrix including mass, retention time and corresponding peak areas of all the detected peaks was obtained.

#### Data Processing and Analysis

The normalized data were imported into SIMCA-P 13.0 (Umetrics, Umea, Sweden) for principal component analysis (PCA) and orthogonal projection to latent structure discrimination analysis (OPLS-DA). The loading plots generated by OPLS-DA were used to screen the key fecal metabolites between the CON and HF groups and between the HF and YCWLP groups, respectively. The key metabolites were selected according to the variable importance in projection (VIP) values (>1.2) and the *p*-values (<0.05), and identified by matching the chemical shift (^1^H NMR) and the m/z (UPLC-MS) with the available databases such as HMDB (http://www.hmdb.ca), KEGG (http://www.genome.jp), METLIN (http://metlin.scripps.edu) and MassBank (http://www.massbank.jp) and the related literature (Chen et al., 2018; Santiago et al., 2019; Liu et al., 2020). In addition, the 7-fold cross-validation method and permutation test (999 permutations) were used to validate the models used in this study. Finally, the key metabolites were imported into MetaboAnalyst 5.0 (http://metpa.metabolomics.ca) for metabolic pathways analysis.

### 16S rRNA Gene Sequencing Analysis

Fecal samples were sent to Beijing Microread Genetics Co., Ltd (Beijing, China) for 16S rRNA gene sequencing. Total genomic DNA was extracted using the QIAamp Fast DNA Stool Mini Kit (Qiagen, Frankfurt, Germany) according to the manufacturer’s protocols. The concentration of DNA was detected by Nanodrop One (Thermofisher, Waltham, Massachusetts, United States), and the purity was detected by 1% agarose gel electrophoresis. The V3-V4 hypervariable regions of the bacteria 16S rRNA gene were amplified with primers 341F (5′- CCTACGGGNBGCASCAG-3′) and 805R (5′- GACTACNVGGGTATCTAATCC-3′) by Veriti 96 Well Thermal Cycler (Thermofisher, Waltham, Massachusetts, United States). The PCR products were purified by adding an equal volume of MagPure A3 XP (Magen) and quantified by the Qubit® 2.0 Fluorometer (Effendorf, Hamburg, Germany) using KAPA Library Quant (Illumina) DNA Standards and Primer Premix Kit (Kapa Biosystems, Boston, Massachusetts, United States) according to the manufacturer’s protocols. At last, the library was sequenced on the IlluminaHiseq 2,500 (Illumina, San Diego, California, United States) platform (PE250) to generate single-end reads.

The original sequencing data in fastq format were filtered by removing the low-quality base sequence using FASTQC (https://www.bioinformatics.babraham.ac.uk/projects/fastqc/), and then merged by HTQC (Version 1.92.3) to generate high-quality clean reads. Then, the chimera sequences were detected and removed by Mothur (Versions 1.38.1) software to obtain effective tags. The effective tags with at least 97% sequence similarity were clustered using Usearch61 method in QIIME (Version 1.9.1) software to obtain the operation taxonomic units (OTUs). The PyNAST (Python Nearest Alignment Space Termination) method in RDP Classifier (http://sourceforge.net/projects/rdp-classifier/, Version 2.2) was used to compare the representative sequences of each OTU with the GreenGenes database (http://greengenes.secondgenome.com/) to obtain the species classification information. Moreover, the relative abundance of species at each taxonomic level was calculated, including phylum, class, order, family and genus.

Alpha diversities such as Chao1 index, Shannon index, Simpson index, PD_whole_tree and rarefaction curve were calculated by QIIME and displayed by R software (Version 3.2.2) to identify the community richness and diversity. Principal coordinate analysis (PCoA), unweighted pair-group method with arithmetic mean (UPGMA), non-metric multidimensional scaling analysis (NMDS) and analysis of similarities (ANOSIM) were performed to analyze the beta diversity between groups by using QIIME software and ggplot2 package in R software (Version 3.2.2). Welch’s *t*-test (SPSS, Version 19.0) was used to screen species with significant differences at each level between the CON and HF groups and between the HF and YCWLP groups (*p*-value < 0.05), respectively. In addition, the linear discriminant analysis (LDA) effect size measurement (LefSe) analysis based on Kruskal-Wallis rank-sum test and Wilcoxon rank-sum test was conducted to identify the abundant taxonomy with significant differences among the three experimental groups.

### Statistical Analysis

All data were expressed as mean ± standard errors of the means. The multiple organ indices, HF-related cytokines, plasma LPS, Ishak scores and fecal metabolites were analyzed by one-way ANOVA test. In 16S rRNA analysis, Welch’s *t*-test and LefSe analysis were used to screen the species with significant abundance differences between the experimental groups. A *p*-value < 0.05 was considered statistically significant. To calculate the correlation coefficient between the pharmacodynamic data, key fecal metabolites and gut microbiota at the genus level, Spearman’s correlation analysis was performed using SPSS 19.0 software and the corresponding heatmaps were visualized using Origin (Version 2018).

## Results

### Yinchen Wuling Powder Attenuates CCl_4_-Induced Hepatic Fibrosis

**FIGURE 1:**
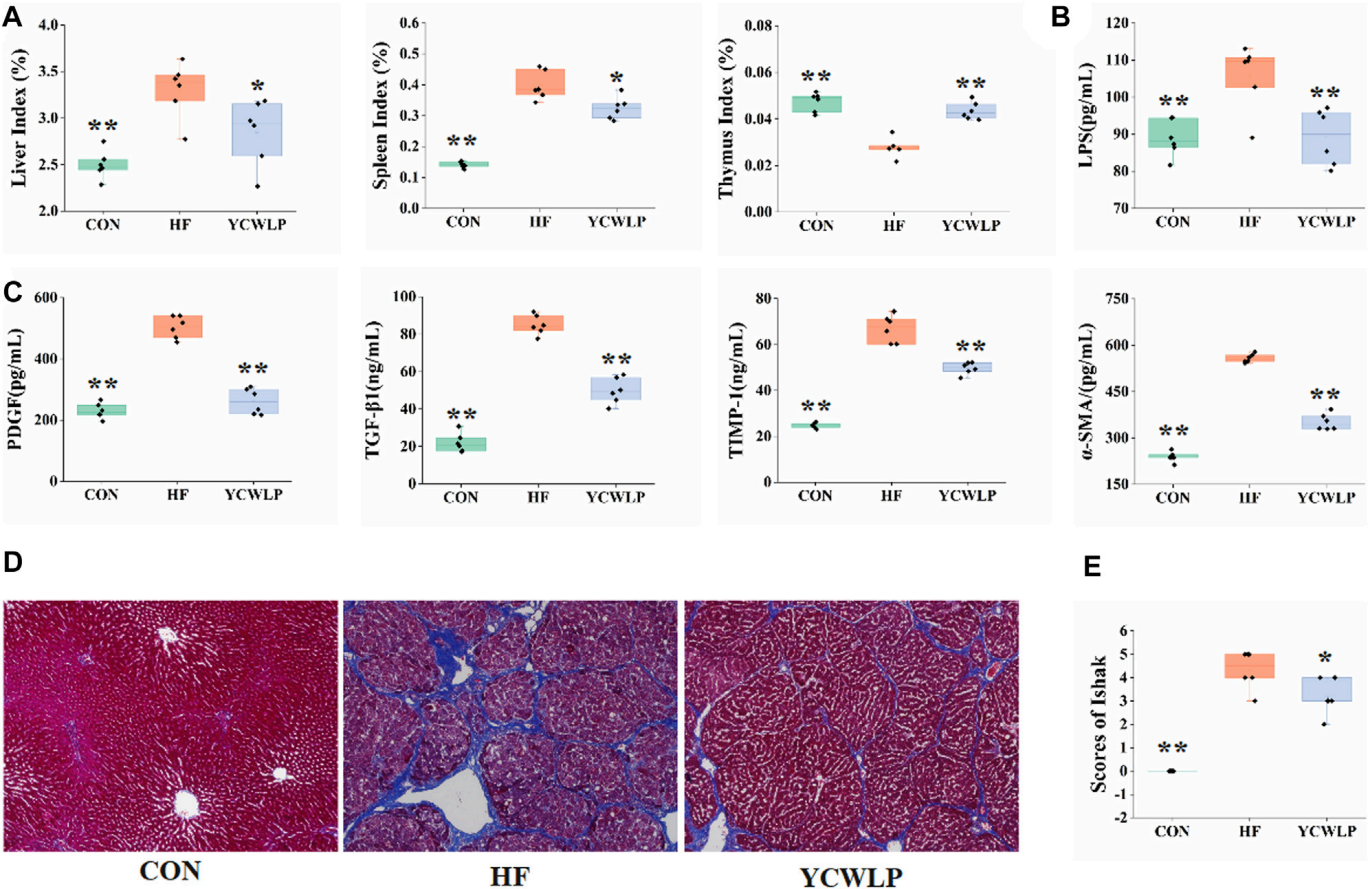
Effect of YCWLP on the CCl_4_−induced HF (*n* = 6). **(A)** Liver, spleen and thymus indices; **(B)** LPS in the plasma samples; **(C)** The expression of PDGF, TGF-β1, TIMP-1 and α-SMA in the liver samples. **(D)** Masson’s trichrome staining of rat liver tissue. Magnification ×100; **(E)** Ishak scores based on Masson’s trichrome staining of rat liver tissue. Significant difference compared with the HF group: * *p*-value < 0.05; ** *p*-value < 0.01.

In a previous study, we investigated the effect of YCWLP on serum AST, ALT, HA, LN, CIV and PIIINP in HF rats (Zhang et al., 2021). The detailed data of these indicators were used for further Spearman’s correlation analysis with gut microbiota in this study (Supplementary Table S1). Furthermore, the effects of YCWLP on multiple organ indices, HF-related cytokines and plasma LPS were also investigated in this study. As the results, the liver index, the spleen index (Figure 1A, *p*-value < 0.01, respectively), the liver levels of PDGF, TGF-β1, TIMP-1 and α-SMA (Figure 1C, *p*-value < 0.01, respectively), and the plasma level of LPS (Figure 1B, *p*-value < 0.01) were significantly increased in the HF group compared with the CON group. And the YCWLP treatment significantly reduced the liver index, the spleen index (Figure 1A, *p*-value < 0.05, respectively), the liver levels of PDGF, TGF-β1, TIMP-1 and α-SMA (Figure 1C, *p*-value < 0.01, respectively), and the plasma level of LPS (Figure 1B, *p*-value < 0.01) in HF rats. However, compared with the CON group, the thymus index of the HF group was significantly decreased (Figure 1A, *p*-value < 0.01). Meanwhile, this change was recovered after the YCWLP treatment (Figure 1A, *p*-value < 0.01). The detailed data of these indices were listed in Supplementary Table S1 for further Spearman’s correlation analysis with gut microbiota.

Masson’s trichrome staining was used to observe the pathological changes of liver tissue among the experimental groups. As shown in Figure 1D, large amounts of collagen fibers proliferated in the portal area of HF rats, with obvious fiber bridging and nodules. After treatment, the proliferation of collagen fibers in the YCWLP group was significantly reduced, and there was occasional fiber bridging between the portal regions. Additionally, the Ishak score analysis of hepatic fibrosis confirmed that the hepatic fibrosis score of the YCWLP group was significantly lower than that of the HF group (Figure 1E). Thus, these results indicated that YCWLP could alleviate the severity of HF in CCl_4_-induced rats.

### Yinchen Wuling Powder Modulated Fecal Metabolism in HF Rats

Representative ^1^H NMR spectra of the fecal samples of the CON, HF and YCWLP groups were shown in Supplementary Figure S1A. 46 endogenous metabolites in the fecal samples were ultimately assigned according to the ^1^H NMR spectra and listed in Supplementary Table S2. Total ion chromatograms (TICs) of each group were overlaid and shown in Supplementary Figure S1B. After normalization of the QC sample data, the extracted ion chromatographic peaks of six ions were selected for method validation. The relative standard deviations (RSDs) of the retention time (RT) and peak areas of the selected ions were calculated and listed in Supplementary Table S3. All values were less than 10%, indicating that the developed method with good repeatability and stability was suitable for the following research.

**FIGURE 2:**
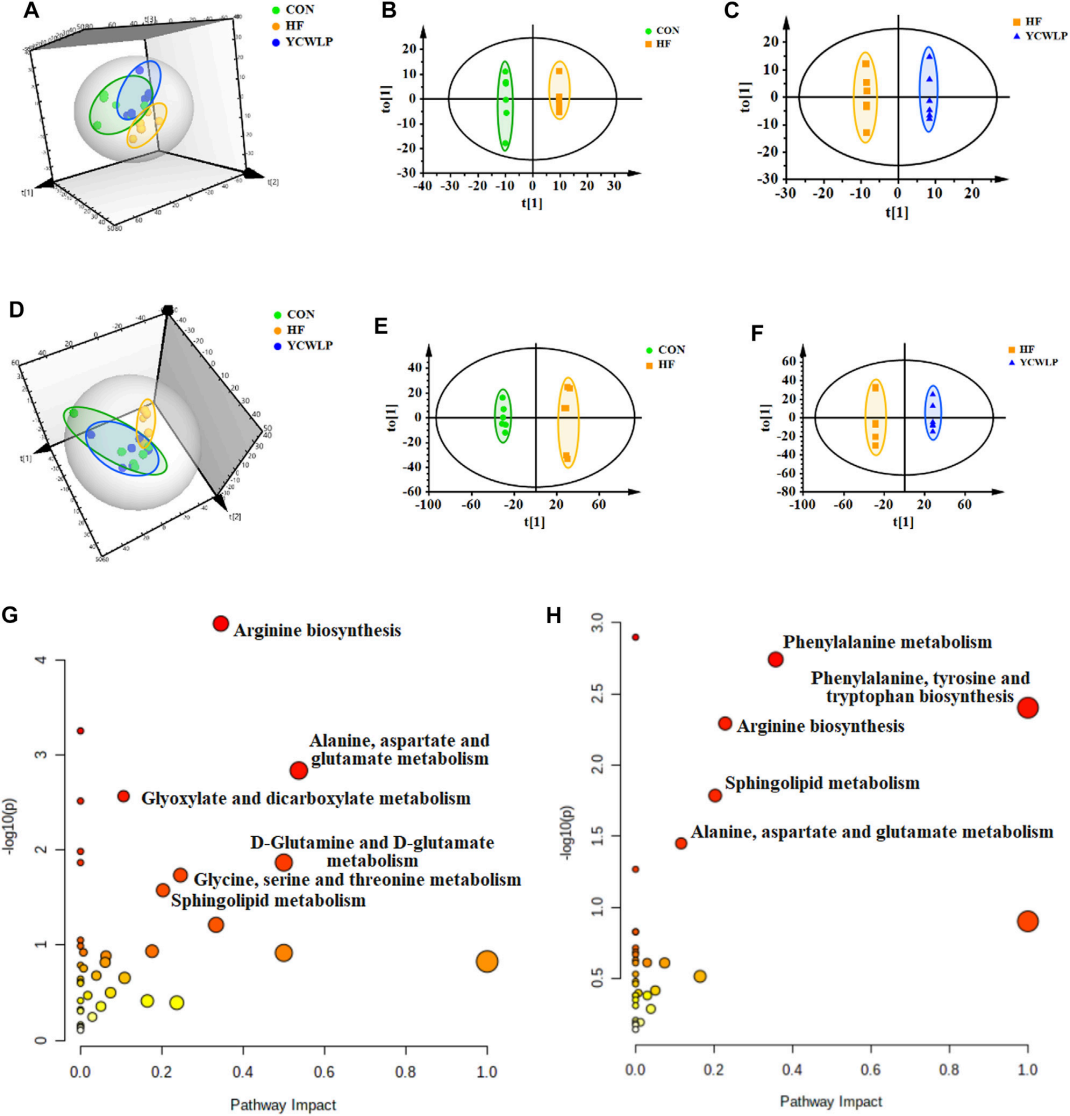
Multivariate data analysis and the metabolic pathway analysis of the fecal samples. 3D PCA score plots of the CON, HF and YCWLP groups based on ^1^H NMR **(A)** and UPLC-MS **(D)**, respectively; OPLS-DA score plots of the CON and HF groups based on ^1^H NMR **(B)** and UPLC-MS **(E)**, respectively; OPLS-DA score plots of the HF and YCWLP groups based on ^1^H NMR **(C)** and UPLC-MS **(F)**, respectively. Meaningful pathway analysis based on the key fecal metabolites between the CON and HF groups **(G)** and between the HF and YCWLP groups **(H)**, respectively (*p*-value < 0.05, impact-value > 0.1).

According to the 3D PCA score plots of both ^1^H NMR and UPLC-MS (Figures 2A,D), the segregations were visible between the CON and HF groups, which indicated that the fecal metabolic profile of HF rats changed significantly. After treatment, the YCWLP group showed a restorable trend and partially coincided with the CON group. In addition, OPLS-DA analysis was also performed to visualize the metabolic alterations occurring between the CON and HF groups and between the HF and YCWLP groups, respectively. As the result, there were significant distinctions between the CON and HF groups (Figures 2B,E) as well as between the HF and YCWLP groups (Figures 2C,F). The above results indicated the hepatoprotective effects of YCWLP against CCl_4_-induced HF, which were consistent with the pharmacodynamic and pathological studies. According to the parameters of 7-fold cross-validation (Supplementary Table S4) and permutations tests of PLS-DA (Supplementary Figures S2E–H), the PCA and OPLS-DA models used in this study were robust.

Based on the loading plots produced by OPLS-DA analysis (Supplementary Figures S2A–D), a total of 57 key metabolites significantly altered between the CON group and the HF group were identified (Supplementary Table S5). Moreover, there were 50 key metabolites significantly changed between the HF group and the YCWLP group (Supplementary Table S6). Among them, 42 fecal metabolites changed due to CCl_4_-treatment could be reversed by YCWLP (Supplementary Table S7), and these metabolites were considered to be the key metabolites of YCWLP affecting HF.

Key metabolites with significant differences between the CON and HF groups and between the HF and YCWLP groups were imported into the MetaboAnalyst 5.0 to screen the meaningful metabolic pathways (*p*-value < 0.05, impact value >0.1), respectively. As shown in Figures 2G,H, three metabolic pathways were selected as the most meaningful metabolic pathways, which were significantly disordered in HF rats and regulated by YCWLP, including arginine biosynthesis, sphingolipid metabolism and alanine, aspartate and glutamate metabolism. Therefore, these three metabolic pathways might be related to the potential mechanism of YCWLP in the treatment of HF.

### 16S rRNA Gene Sequencing Analysis

#### Overall Structural Modulation of Gut Microbiota by Yinchen Wuling Powder Treatment

A total of 337,264 clean reads were obtained from 15 fecal samples (5 in each group), and each sample produced an average of 22,484 ± 788 clean reads. Rarefaction curves of each sample tended to be flat within a certain number of sequences sampled, which indicated that the sequencing depth of each sample in this study was adequate (Supplementary Figure S3). As shown in alpha diversity analysis, the Chao1 and Shannon indices were significantly lower in the HF group than those in the CON group (Supplementary Figure S4A, Table S8, *p*-value < 0.01, respectively), whereas there were no significant differences in the Simpson and PD_whole_tree indices between these two groups. Compared with the HF group, the Shannon index of the YCWLP group was significantly increased (Supplementary Figure S4A, Table S8, *p*-value < 0.05). The results indicated that the richness and diversity of the gut microbiota were significantly reduced after CCl_4_ treatment, which was consistent with the previous research (Li et al., 2020), and the YCWLP treatment could significantly improve the gut microbiota diversity of HF rats.

Then, unweighted UniFrac-based-PCoA, NMDS and UPGMA were performed to analyze the changes in gut microbiota community structures among different groups. As shown in PCoA analysis, the gut microbiota profiles of the CON, HF and YCWLP groups were separated obviously with the total variances of PC1 16% and PC2 11% (Supplementary Figure S4B). The NMDS analysis depicted that the HF group was clearly separated from the CON group, and the YCWLP group was located in the middle of them and showed a trend away from the HF group and close to the CON group (Supplementary Figure S4D). The stress value was less than 0.2, which indicated that the NMDS analysis was able to accurately reflect the difference between samples (Clarke, 1993). Moreover, the hierarchical clustering analysis of UPGMA also manifested that the distance between the YCWLP group and the CON group was shorter than that between the YCWLP group and the HF group (Supplementary Figure S4C). The results above indicated that YCWLP could ameliorate the dysbiosis of the gut microbiota induced by CCl_4_. In addition, according to the ANOSIM analysis (Supplementary Figure S4E), the results of R-value > 0, *p* < 0.01 showed that the difference between the groups was significantly greater than that within the group, indicating that the grouping of this study was meaningful.

#### Differential Gut Microbiota in Hepatic Fibrosis and Yinchen Wuling Powder-Treated Rats

The top ten gut microbiota in the relative abundance at the phylum, class, order and family levels were shown in Supplementary Figure S5A and the species with significant changes at each level were evaluated and shown in Supplementary Figure S5B. At the phylum level, compared with the CON group, the relative abundance of *Firmicutes*, *Chloroflexi*, *Gemmatimonadetes*, TM7 and *Tenericutes* of the HF group were significantly increased, while the relative abundance of *Bacteroidetes* was significantly decreased. Compared with the HF group, YCWLP treatment could significantly restore the relative abundance of *Gemmatimonadetes*. At the class level, the relative abundance of *Acidobacteria-6*, *Acidimicrobiia*, *Ktedonobacteria*, TM7-1, *Gemmatimonadetes*, *Clostridia*, *Deltaproteobacteria,* TM7-3 and *Mollicutes* in the HF group were significantly higher than those in the CON group, whereas *Bacteroidia* and *Actinobacteria* were significantly lower. After treatment, the relative abundance of *Acidobacteria-6, Acidimicrobiia, Ktedonobacteria*, TM7-1 and *Gemmatimonadetes* in the YCWLP group were significantly restored. At the order level, the relative abundance of *Bacteroidales* and *Actinomycetales* were significantly lower and the relative abundance of *Acidimicrobiales*, *Clostridiales*, *Desulfovibrionales,* CW040 and RF39 were significantly higher in the HF group than those in the CON group. In addition, the relative abundance of *Acidimicrobiales* was significantly reversed by YCWLP treatment. At the family level, the reduced abundance of Prevotellaceae, Micrococcaceae, Streptococcaceae and Peptococcaceae, and the increased abundance of Ruminococcaceae, [Barnesiellaceae], Dehalobacteriaceae, F16, Desulfovibrionaceae and Thermomonosporaceae were observed in HF rats. Compared with the HF group, YCWLP significantly restored the relative abundance of Thermomonosporaceae in HF rats. Interestingly, the relative abundance of *Bifidobacteriales* at the order level and Bifidobacteriaceae at the family level were significantly increased after YCWLP treatment.

**FIGURE 3:**
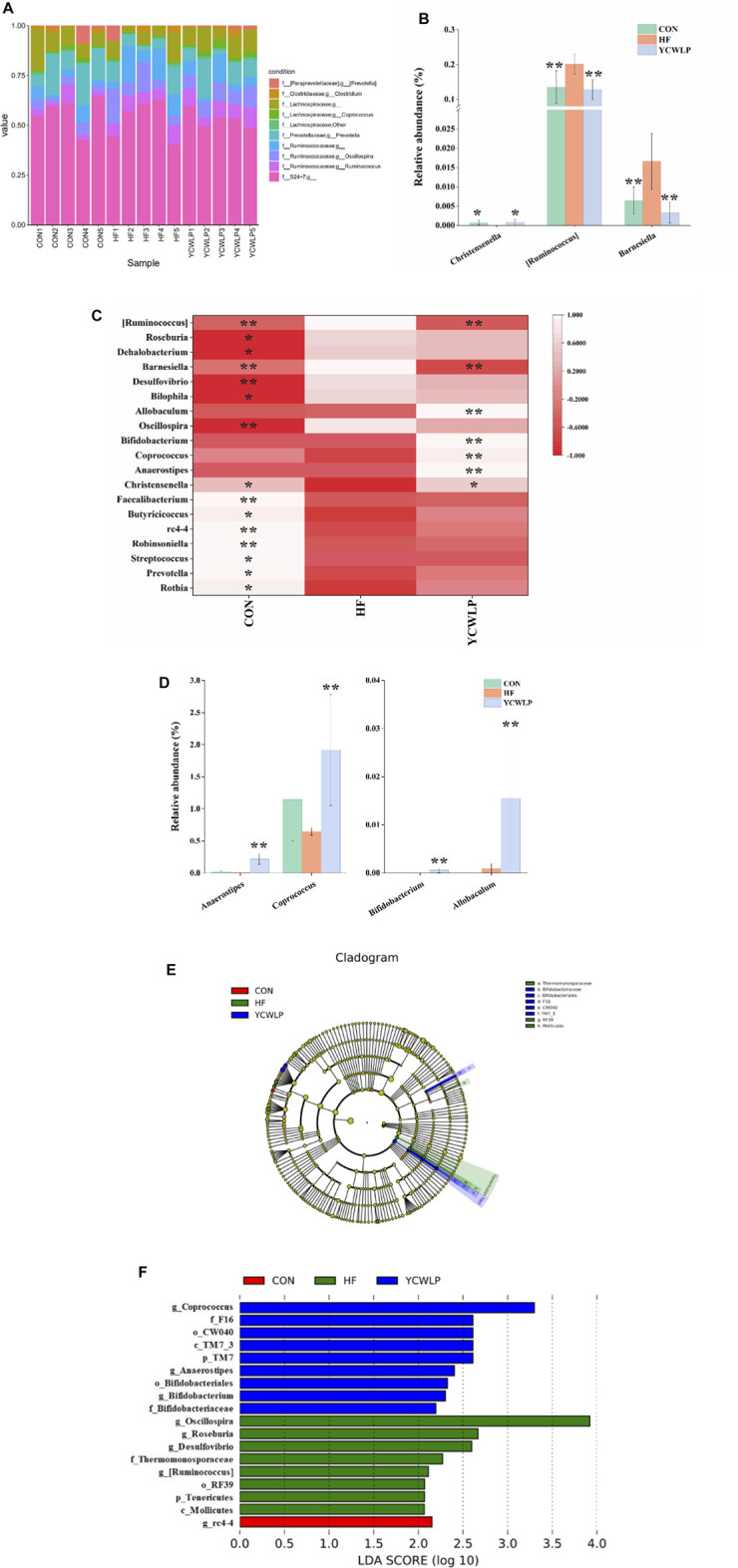
Differences of gut microbiota composition among the CON, HF and YCWLP groups. **(A)** Top ten gut microbiota in the relative abundance at the genus level; **(B)** The relative abundance of *Christensenella* [*Ruminococcus*] and *Barnesiella*; **(C)** Heatmap of genera with significant variations; **(D)** The relative abundance of *Anaerostipes*, *Coprococcus*, *Bifidobacterium* and *Allobaculum*; **(E)** Cladogram generated by the LEfSe analysis; **(F)** Linear discriminant analysis (LDA) scores of the discriminative taxa. The letters p, c, o, f and g represented phylum, class, order, family and genus, respectively. Significant difference compared with the HF group: * *p*-value < 0.05; ** *p*-value < 0.01.

At the genus level, the top ten genera in the relative abundance were shown in Figure 3A. The Welch’s *t*-test showed that a total of 19 genera changed significantly among the experimental groups (Figure 3C), and their specific relative abundance was shown in Supplementary Figure S6. Among them, 15 genera were significantly changed due to the CCl_4_ treatment. In detail, we observed significantly decreased abundance of *Rothia*, *Prevotella*, *Streptococcus*, *Robinsoniella*, rc4-4, *Butyricicoccus*, *Faecalibacterium* and *Christensenella*, and significantly increased abundance of *Oscillospira, Bilophila, Desulfovibrio, Barnesiella*, *Dehalobacterium*, *Roseburia* and [*Ruminococcus*] in HF rats compared with the CON rats. After treatment, three genera including *Christensenella* [*Ruminococcus*] and *Barnesiella* were significantly reversed by YCWLP (Figure 3B). In addition, YCWLP treatment also significantly increased the relative abundance of *Anaerostipes, Coprococcus*, *Bifidobacterium* and *Allobaculum* in HF rats (Figure 3D).

The LEfSe analysis, which emphasizes the statistical significance and biological correlation, was also performed to search for biomarkers with statistical significance among the CON, HF and YCWLP groups (Figures 3E,F). In this study, the discriminative features of the bacterial taxa were identified with an LDA score >2.0. According to the ranked bacterial taxa, the HF rats were enriched with p_Tenericutes, c_Mollicutes, o_RF39, f_Thermomonosporaceae, g_Oscillospira, g_Roseburia, g_Desulfovibrio and g_[Ruminococcus], which suggested that the changes of these gut microbiota might promote the deterioration of HF. After treatment, the rats of the YCWLP group were enriched with p_TM7, c_TM7_3, o_CW040, o_Bifidobacteriales, f_F16, f_Bifidobacteriaceae, g_Coprococcus, g_Anaerostipes and g_Bifidobacterium. Combined with the differential gut microbiota analyzed by Welch’s *t*-test at the genus level (Figures 3B,D), it can be concluded that [*Ruminococcus*] played the most significant role in YCWLP treatment. We also noticed that *Bifidobacterium* as a well-known probiotic was significantly increased after YCWLP treatment, together with *Coprococcus* and *Anaerostipes* which were the characteristic genera for the rats of the YCWLP group, might also be responsible for YCWLP treatment.

### Hepatic Fibrosis-Related Genera Regulated by Yinchen Wuling Powder

**FIGURE 4:**
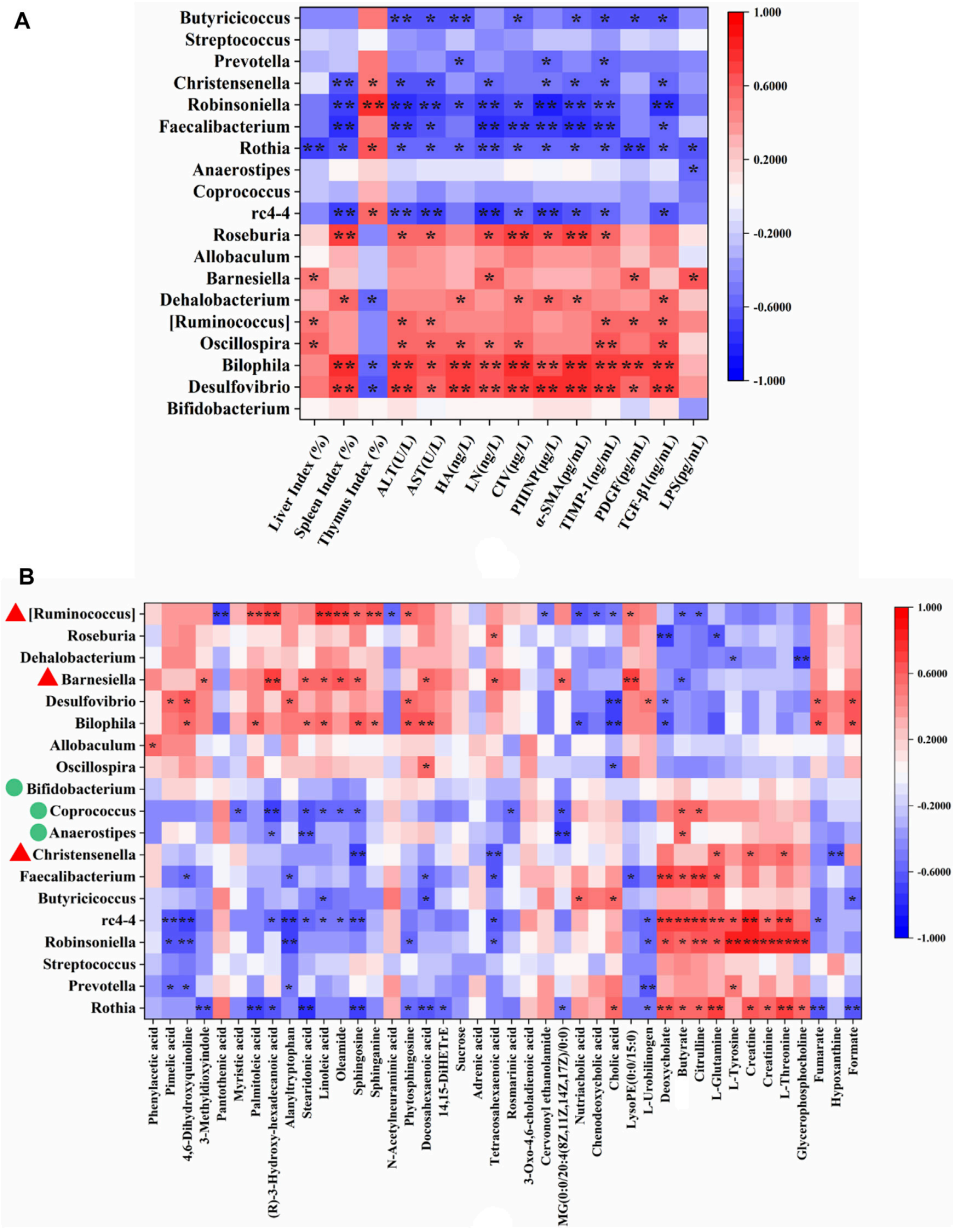
Spearman’s correlation analysis of HF-related pathological indices, key fecal metabolites and significantly changed genera. **(A)** Correlations between 19 significantly changed genera and 14 HF-related pathological indices; **(B)** Correlations between 19 significantly changed genera and 42 key fecal metabolites reversed by YCWLP treatment. ^*^|r| > 0.5 and *p*-value < 0.05; ^**^|r| > 0.5 and *p*-value < 0.01. The genera marked with red triangles in **(B)** represent the disordered HF-related genera reversed by YCWLP treatment. The genera marked with green circles in **(B)** represent the genera increased significantly after YCWLP treatment.

The correlations between 19 genera that changed significantly among the experimental groups (Figure 3C) and 14 HF-related pathological indices (Supplementary Table S1) were conducted by Spearman’s correlation analysis. The results were summarized in Supplementary Table S9 and presented as a heatmap (Figure 4A). In general, there were 13 genera closely related to the phenotype of HF (≥4 pathological indices were closely correlated with certain genus). Among them, *Desulfovibrio*, *Bilophila*, *Oscillospira* [*Ruminococcus*], *Dehalobaterium*, *Barnesiella* and *Roseburia* showed significant positive correlations with the pathological changes, while *rc4-4*, *Rothia*, *Faecalibacterium*, *Robinsoniella*, *Christensenella* and *Butyricicoccus* showed significant negative correlations. Of these 13 genera, *Christensenella, [Ruminococcus]* and *Barnesiella* could be significantly reversed by YCWLP in HF rats. Therefore, these three genera might be the targets of YCWLP in the treatment of CCl_4_-induced HF.

### Correlations Between Gut Microbiota and Fecal Metabolites

Spearman’s correlation analysis was also conducted to analyze the correlations between the 19 significant changed genera and 42 key fecal metabolites that could be reversed by YCWLP (Supplementary Table S7). As shown in Figure 4B and Supplementary Table S9, [*Ruminococcus*] showed a negative correlation with citrulline and positive correlations with the metabolites related to sphingolipid metabolism. *Christensenella* was negatively correlated with sphingosine and positively correlated with L-Glutamine. *Barnesiella* was positively correlated with sphingosine.

## Discussion

In this study, we further verified the therapeutic effect of YCWLP on HF using Masson’s trichrome staining. Additionally, the effects of YCWLP on multiple organ indices, HF-related cytokines and plasma LPS in HF rats were also investigated, so as to provide evidence for the exploration of the therapeutic mechanism of YCWLP. The results showed decreased levels of the liver and spleen indices and an increased level of the thymus index following YCWLP treatment. The spleen is mainly involved in humoral immunity, while the thymus is mainly involved in cellular immunity. The callback effect of YCWLP on these indices reflected its hepatoprotective and immunomodulatory effects. Compared with the HF group, YCWLP could significantly reduce the expression of PDGF, TGF-β1, TIMP-1 and α-SMA in the liver. These cytokines mainly participate in the activation of hepatic stellate cells (HSCs), and the production and degradation of extracellular matrix (ECM), which indicated that the anti-fibrosis mechanism of YCWLP might be related to inhibiting HSC activity and reducing ECM synthesis. In addition, YCWLP could restore the level of LPS in plasma of HF rats, indicating that YCWLP could protect the intestinal tract and reduce the release of LPS from liver cells into blood circulation.

It has been proved that the therapeutic mechanism of YCWLP on HF is related to gut microbiota (Zhang et al., 2021). In this study, multiomics was used to investigate the effect of YCWLP on gut microbiota in HF rats and the interaction between gut microbiota and host metabolism. 16S rRNA sequencing showed that the diversity and richness of gut microbiota in HF rats were both significantly decreased. YCWLP treatment could significantly improve the diversity of gut microbiota in rats with HF, but had no significant effect on the richness. PCoA and NMDS analysis indicated significant differences in gut microbiota composition among the CON, HF and YCWLP groups. In UPGMA analysis, the beta diversity of the YCWLP-treated rats showed a greater similarity to the rats in the CON group than those in the HF group. Spearman’s correlation analysis was performed on the 19 genera with significant changes among the experimental groups and 14 HF-related pathological indices. Finally, 13 genera were identified to be intimately related to HF phenotype, from which strong positive relationships were identified for *Desulfovibrio*, *Bilophila*, *Oscillospira* [*Ruminococcus*], *Dehalobaterium*, *Barnesiella* and *Roseburia,* while significant negative relationships were identified for *rc4-4*, *Rothia*, *Faecalibacterium*, *Robinsoniella*, *Christensenella* and *Butyricicoccus. Desulfovibrio* is a sulfate-reducing bacteria, which has been proved to promote the occurrence and development of HF (Chen, 2018). *Bilophila* is significantly associated with fatty liver disease caused by overfeeding. High-fat diet can increase its proportion in gut microbiota and increase the risk of inflammatory bowel disease and hepatobiliary disease (Jian et al., 2021). Moreover, both *Desulfovibrio* and *Bilophila* can produce LPS and promote inflammation (Wei et al., 2015; Song et al., 2017). Studies have shown that [*Ruminococcus*] is significantly positively correlated with the degree of HF (Boursier et al., 2016). In addition, the up-regulation of [*Ruminococcus*] can be used as the intestinal microbiological characteristics of the progress of non-alcoholic fatty liver disease (NAFLD) (Del Chierico et al., 2017). *Barnesiella* has been reported to be positively correlated with glucose and lipid metabolism disorders and dyslipidemia (Deng et al., 2020). Furthermore, *Barnesiella* was significantly enriched in hepatocellular carcinoma (HCC) patients, and its relative abundance gradually increased during the development of HCC (Jiang et al., 2020). The relative abundance of these pathogens increased significantly in HF rats, while the relative abundance of beneficial bacteria *Christensenella* and short-chain fatty acid producing bacteria *Butyricicoccus* and *Faecalibacterium* decreased significantly. The changes of these gut microbiota might promote the deterioration of HF.

**FIGURE 5:**
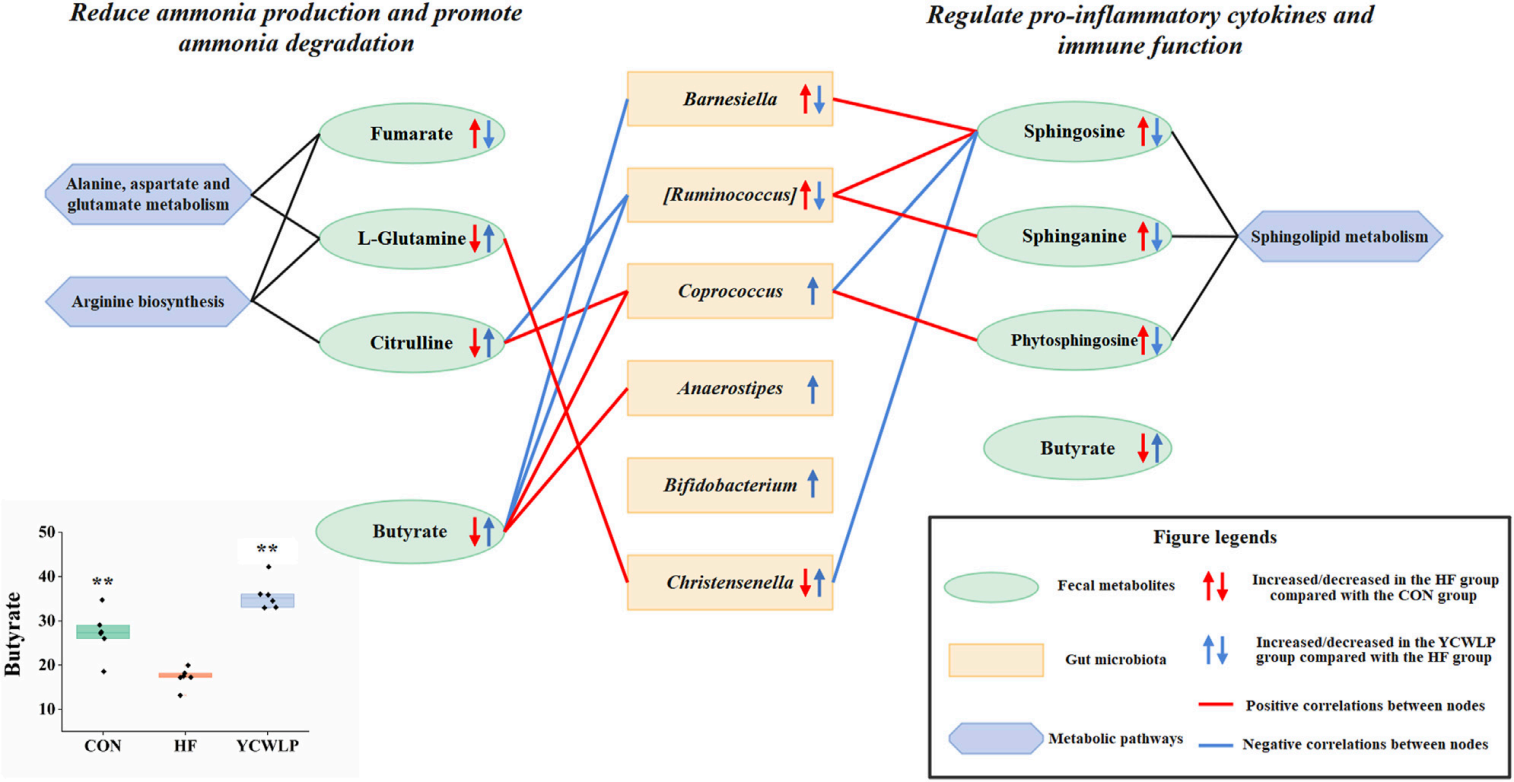
Mechanism of YCWLP in the treatment of HF based on the interactions between gut microbiota and host metabolism. Significant difference compared with the HF group: * *p*-value < 0.05; ** *p*-value < 0.01.

YCWLP treatment could significantly restore the relative abundance of *Christensenella* [*Ruminococcus*] and *Barnesiella* in HF rats. Therefore, it can be considered that these three genera were responsible for the treatment of YCWLP. In addition, LEfSe analysis showed that *Bifidobacterium*, *Coprococcus* and *Anaerostipes* were the characteristic genera enriched in the YCWLP group (LDA >2). The relative abundance of these three genera in YCWLP-treated rats were much higher than those in the CON and HF groups, which might be the reason why they had no significant correlations with the HF phenotype. However, they might still play a key role in the treatment of HF with YCWLP. Interestingly, we also found that these three genera are closely related to the production of butyrate. *Coprococcus* and *Anaerostipes* belonging to Lachnospiraceae are butyrate-producing bacteria (Feng et al., 2018). The main metabolites of *Bifidobacterium* are acetic acid, lactic acid and formic acid. In the intestinal ecosystem, lactic acid and acetate are used to produce butyrate or propionate by *Firmicutes* (Scott et al., 2014). The results of this study also showed that the content of butyrate in feces increased significantly after YCWLP treatment (Figure 5), and *Coprococcus* and *Anaerostipes* were strongly positively correlated with butyrate (Figure 4B). Butyrate is an important energy source of intestinal epithelial cells, which can protect the intestinal barrier by stimulating tight junction and mucus formation (Usami et al., 2015). Butyrate can also inhibit the proliferation of pathogenic bacteria such as *Escherichia coli*, *Staphylococcus* and *Clostridium* by releasing H^+^, and promote the growth and reproduction of beneficial bacteria *Lactobacillus* (Allen et al., 2018). Studies have shown that the decrease of Lachnospiraceae, which are characterized by butyrate production, may lead to an increase of colonic pH. This is believed to promote the production and absorption of ammonia, and resulting in hepatic encephalopathy (Woodhouse et al., 2018). In addition, butyrate can also regulate the immune response by affecting the production of inflammatory mediators, the migration and adhesion of immune cells and cell function (Correa et al., 2016). Therefore, the effect of YCWLP on increasing the production of butyrate is of great significance to improve HF.

**FIGURE 6:**
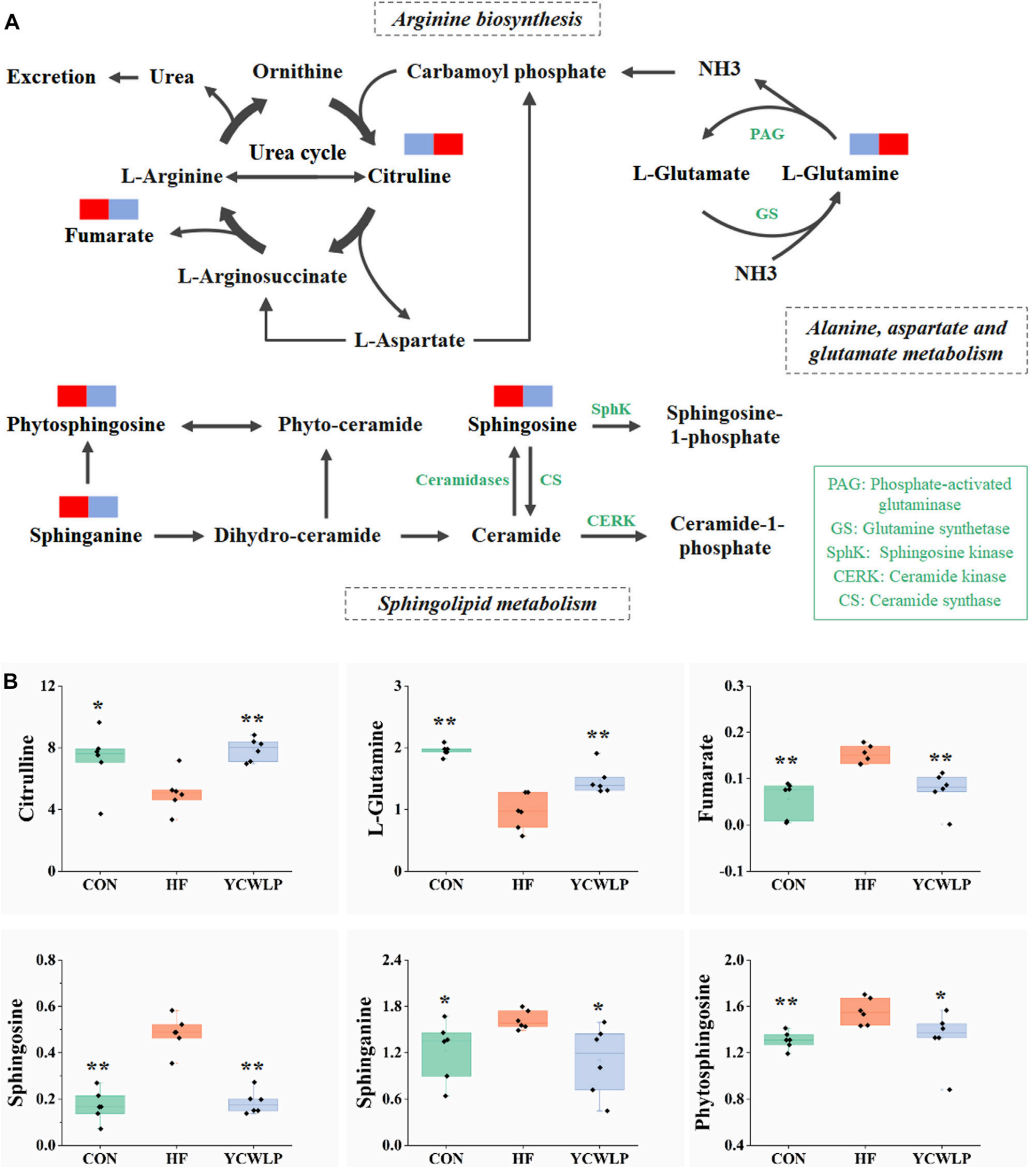
Schematic of arginine biosynthesis, sphingolipid metabolism and alanine, aspartate and glutamate metabolism pathways **(A)** and the related metabolites **(B)**. The blue rectangles in **(A)** represent being significantly down-regulated, and the red rectangles represent being significantly up-regulated. Significant difference compared with the HF group: * *p*-value < 0.05; ** *p*-value < 0.01.

Fecal non-targeted metabolomics showed that YCWLP could reverse 42 metabolites that were changed by CCl_4_ in HF rats. Through metabolic pathway analysis, we found that YCWLP could significantly correct the disorder of three metabolic pathways, including arginine biosynthesis, sphingolipid metabolism and alanine, aspartate and glucose metabolism. The relationship between the potential metabolic pathways and their corresponding metabolites were shown in Figure 6. Intestinal flora affects host metabolism by transforming, absorbing and metabolizing exogenous substances (Li et al., 2019). The changes of metabolites reflect the alternations of gut microbiota to a certain extent. Therefore, in order to explore the mechanism of gut microbiota regulating the occurrence and development of HF and the treatment of YCWLP through affecting host metabolism, we conducted Spearman’s correlation analysis on 42 key fecal metabolites and 19 intestinal microflora, which were related to the treatment of HF with YCWLP (Figure 4B).

### Amino Acid Metabolism

Citrulline is the intermediate of the urea cycle, and the urea cycle is the main way to eliminate ammonia. Most of the ammonia in the human body is produced and absorbed in the gastrointestinal tract (Romero-Gomez et al., 2009). After absorption, most of the ammonia in the portal vein is detoxified through the urea cycle, and the rest is removed by glutamine synthetase in liver cells around the vein, which catalyzes the reaction of ammonia with glutamate to produce glutamine (Wright et al., 2011). In the condition of HF, the function of urea cycle is weakened, resulting in the accumulation of ammonia (Dabos et al., 2015). On the other hand, liver injury can lead to the dysfunction of glutamine synthetase, which can not effectively catalyze the combination of glutamate and ammonia, leading to the increase of blood ammonia. Clinical studies have shown that reduction of urea production and hyperammonemia are characteristic phenomena in patients with HF (Weissenborn et al., 2007). In this study, the levels of citrulline and glutamine in feces of rats with HF were significantly decreased, indicating that the urea cycle was weakened and the ammonia clearance pathway was blocked. After YCWLP intervention, the levels of citrulline and glutamine were significantly increased (Figure 6B), indicating YCWLP could improve the urea cycle, promote glutamine synthesis and reduce ammonia accumulation.

In addition, the small intestinal bacterial overgrowth and the damage of the intestinal mucosal barrier caused by HF will also lead to the increase of ammonia production, and a large amount of ammonia will be absorbed into the blood circulation, resulting in the accumulation of blood ammonia. The metagenomic experiment confirmed that the genes related to ammonia metabolism and amino acid transport in the gut microbiota of patients with HF were highly expressed (Qin et al., 2014). In this study, Spearman’s correlation analysis showed that citrulline was negatively correlated with [*Ruminococcus*], and positively correlated with *Rothia*, *Robinsoniella*, *rc4-4*, *Faecalibacterium* and *Coprococcus*. Glutamine was positively correlated with *Rothia*, *Robinsoniella*, *Faecalibacterium*, *rc4-4* and *Christensenella*, and negatively correlated with *Roseburia.* Among these genera, YCWLP could significantly affect the relative abundance of [*Ruminococcus*], *Coprococcus* and *Christensenella.* In 16Sr RNA sequencing analysis [*Ruminococcus*] was the most critical genus in the treatment of HF with YCWLP, which was significantly enriched in HF rats. Ammonia is the necessary nitrogen source for the growth and reproduction of [*Ruminococcus*]. Therefore, we speculated that the accumulation of ammonia in the intestine of HF rats might be responsible for its enrichment. However, YCWLP treatment could significantly reduce the abundance of [*Ruminococcus*], which might be related to its effect of reducing ammonia accumulation. As mentioned before, *Coprococcus* was the characteristic genus of YCWLP-treated rats, which could reduce ammonia production and absorption by producing butyrate.

In summary, YCWLP could promote the production of butyrate to reduce the production and absorption of ammonia by improving the abundance of butyrate-producing bacteria. On the other hand, YCWLP could accelerate ammonia elimination by promoting urea cycle and glutamine synthesis.

### Sphingolipid Metabolism

Sphingolipids (SLs), as a class of bioactive lipids, are the main structural components of the cell membrane (Kraft, 2017). SLs mainly include ceramide, sphingosine and sphingosine-1-phosphate, which participate in the occurrence and development of liver diseases by regulating cell proliferation and differentiation, gene expression and apoptosis (Ilan, 2019). Studies have shown that liver injury can lead to the disturbance of sphingosine metabolism and significant changes of the related substances (Acunha et al., 2018). In this study, the levels of sphingosine, sphinganine and phytosphingosine in the feces of HF rats were significantly increased (Figure 6B), indicating that the metabolism of sphingolipid was disordered. In the organism, sphingosine, sphinganine and phytosphingosine play an important role in ceramide production (Figure 6A). Ceramide has been proved to be implicated in inflammation, which can attenuate LPS response in macrophages and regulate T cell function (Ali et al., 2015). Inhibition of ceramide synthesis may affect pro-inflammatory cytokine signaling and immune cell chemotaxis to a certain extent, further aggravating the liver injury (Montefusco et al., 2018). YCWLP treatment could significantly restore the levels of sphingosine, sphinganine and phytosphingosine (Figure 6B), indicating that YCWLP could regulate the disorder of sphingosine metabolism in HF rats, and treat HF by regulating pro-inflammatory cytokines and immune function.

Spearman’s correlation analysis showed that sphingosine was positively correlated with *Bilophila*, *Barnesiella* and [*Ruminococcus*], while negatively correlated with *Rothia*, *rc4-4*, *Christensenella* and *Coprococcus*. Sphinganine was positively correlated with *Bilophila* and [*Ruminococcus*]. Phytosphingosine was positively correlated with *Bilophila*, *Desulfovibrio* and [*Ruminococcus*], while negatively correlated with *Rothia* and *Robinsoniella*. Given these correlations, it’s likely the modulatory effects of YCWLP on sphingolipid metabolism might occur by affecting the relative abundances of *Barnesiella*, [*Ruminococcus*], *Christensenella*, *Coprococcus* and *Anaerostipes*.

## Conclusion

In this study, an integrated method of 16S rRNA gene sequencing combined with ^1^H NMR and UPLC-MS based metabolomics was performed to evaluate the effects of YCWLP on the gut microbiota and the interaction between gut microbiota and host metabolism in rats with CCl_4_-induced HF. The therapeutic mechanisms of YCWLP on HF were likely linked to restoring the dysbiosis of *Barnesiella* [*Ruminococcus*] and *Christensenella*, increasing the relative abundance of *Bifidobacterium*, *Coprococcus* and *Anaerostipes* which closely related to butyrate production, and regulating the disorder of arginine biosynthesis, sphingolipid metabolism and alanine, aspartate and glutamate metabolism in HF rats (Figure 5). These regulatory effects suggested that YCWLP might treat HF by reducing ammonia production and promoting ammonia degradation, regulating pro-inflammatory cytokines and immune function. Moreover, we found that butyrate plays a pivotal role in the treatment of HF by YCWLP. Increasing the abundance of butyrate-producing bacteria might be an important therapeutic mechanism of YCWLP on HF. Our study focuses on the interaction mechanism between gut microbiota and host metabolisms, which provides new insights into the potential anti-fibrosis mechanism of YCWLP and lays a foundation for further development of YCWLP as a potential strategy in treating HF. However, although non-targeted metabolomics analysis covers a wide range of endogenous substances, it still has the weakness of low sensitivity and selectivity. Besides, 16S rRNA analysis lacks the ability to predict the function of gut microbiota. Therefore, the detailed modulatory effects of YCWLP on gut microbiota and fecal metabolism needs to be further investigated by metagenomics and targeted metabolomics.

## Data Availability

The data generated in this article can be found in NCBI using accession PRJNA732726.
