# Supplementary material for: Comprehensive Analysis of Fecal Microbiome and Metabolomics in Hepatic Fibrosis Rats Reveal Hepatoprotective Effects of Yinchen Wuling Powder From the Host-Microbial Metabolic Axis
Source: Front Pharmacol. 2021 Jul 27;12:713197. doi: 10.3389/fphar.2021.713197 (PMC8353151; doi:10.3389/fphar.2021.713197)
Supplement: Supplementary file 1 [file DataSheet1.doc]

***Supplementary materials***

**Table S1** Changes in HF-related pathological indices after YCWLP treatment

|  | CON | HF | YCWLP |
| --- | --- | --- | --- |
| ALT (U/L) | 54.27 ± 13.55^**^ | 800.96 ± 110.14 | 400.23 ± 99.19^**^ |
| AST (U/L) | 124.13 ± 39.44^**^ | 1555.04 ± 261.12 | 756.99 ± 160.89^**^ |
| HA (ng/L) | 238.26 ± 22.45^**^ | 337.31 ± 16.35 | 293.16 ± 10.52^**^ |
| LN (μg/L) | 220.65 ± 15.48^**^ | 459.82 ± 28.41 | 316.59 ± 53.15^**^ |
| CIV (μg/L) | 28.59 ± 2.74^**^ | 80.29 ± 4.10 | 53.37 ± 3.43^**^ |
| PⅢNP (μg/L) | 9.61 ± 0.91^**^ | 17.07 ± 1.05 | 12.37 ± 0.77^**^ |
| Liver Index (%) | 2.50 ± 0.14^**^ | 3.30 ± 0.27 | 2.85 ± 0.32^*^ |
| Spleen Index (%) | 0.14 ± 0.0091^**^ | 0.40 ± 0.042 | 0.32 ± 0.033^*^ |
| Thymus Index (%) | 0.047 ± 0.0037^**^ | 0.028 ± 0.0037 | 0.044 ± 0.0034^**^ |
| α-SMA (pg/mL) | 238.49 ± 14.84^**^ | 557.59 ± 12.84 | 351.60 ± 23.93^**^ |
| TIMP-1 (ng/mL) | 24.66 ± 1.05^**^ | 66.99 ± 5.40 | 49.71 ± 2.35^**^ |
| PDGF (pg/mL) | 230.03 ± 22.80^**^ | 503.65 ± 33.15 | 261.45 ± 38.19^**^ |
| TGF-β1(ng/mL) | 21.99 ± 4.63^**^ | 85.00 ± 4.79 | 49.83 ± 6.36^**^ |
| LPS (pg/mL) | 88.88 ± 4.52^**^ | 105.79 ± 8.13 | 89.18 ± 6.90^**^ |

Significant difference compared with the HF group: ^*^ *p*-value < 0.05; ^**^ *p*-value < 0.01.

**Table S2** Endogenous metabolites in the fecal samples based on ^1^H NMR spectra

| No. | Metabolites | Chemical shift (ppm) | No. | Metabolites | Chemical shift (ppm) |
| --- | --- | --- | --- | --- | --- |
| 1 | Deoxycholate | 0.68 (s) | 24 | Creatine | 3.04 (s), 3.95 (s) |
| 2 | Butyrate | 0.92 (t), 1.56 (m), 2.16 (t) | 25 | L-Tyrosine | 3.08 (m), 3.92 (m), 6.90 (d), 7.20 (d) |
| 3 | L-Isoleucine | 0.94 (t) ,1.01 (d), 3.65 (d) | 26 | Malonate | 3.12 (s) |
| 4 | L-Leucine | 0.97 (d) | 27 | Choline | 3.20 (s) |
| 5 | L-Valine | 0.99 (d), 3.60 (d) | 28 | Taurine | 3.25 (t), 3.44 (t) |
| 6 | Propionic acid | 1.06 (t), 2.19 (q) | 29 | TMAO | 3.27 (s) |
| 7 | α-Ketoisovalerate | 1.13 (d), 3.02 (m) | 30 | Methanol | 3.37 (s) |
| 8 | L-Threonine | 1.33 (d), 3.59 (d), 4.26 (m) | 31 | Phenylacetate | 3.54 (s), 7.32 (m), 7.37 (m) |
| 9 | Lactate | 1.34 (d), 4.13 (q) | 32 | Glycine | 3.57 (s) |
| 10 | L-Alanine | 1.48 (d) | 33 | Glycerol | 3.68 (dd) |
| 11 | Cadaverine | 1.48 (d), 1.72 (m), 3.02 (t) | 34 | Hippurate | 3.99 (d) |
| 12 | Citrulline | 1.57 (m) | 35 | Creatinine | 4.08 (s) |
| 13 | L-Lysine | 1.73 (m), 3.77 (t) | 36 | Glycerophosphocholine | 4.32 (dd) |
| 14 | Acetate | 1.92 (s) | 37 | β-Glucose | 4.66 (d) |
| 15 | L-Proline | 2.05 (m), 2.36 (m), 3.34 (m), 4.11 (m) | 38 | α-Glucose | 5.24 (d) |
| 16 | L-Glutamate | 2.08 (m), 2.36 (m), 3.75 (m) | 39 | Uracil | 5.81 (d), 7.54 (d) |
| 17 | Methionine | 2.13 (m), 2.14 (s) | 40 | Fumarate | 6.52 (s) |
| 18 | N-acetyl-5-aminosalicylate | 2.18 (s) | 41 | L-Phenylalanine | 7.33 (m), 7.38 (m), 7.43 (m) |
| 19 | Succinate | 2.41 (s) | 42 | Histidine | 7.90 (s) |
| 20 | L-Glutamine | 2.48 (m) | 43 | Adenine | 8.14 (s) |
| 21 | Dimethylamine (DMA) | 2.71 (s) | 44 | Hypoxanthine | 8.19 (s), 8.22 (s) |
| 22 | L-Aspartate | 2.80 (dd) | 45 | Adenosine | 8.27 (s), 8.38 (s) |
| 23 | Cysteine | 3.04 (m) | 46 | Formate | 8.46 (s) |

**Table S3** Stability and repeatability results of the UPLC-MS platform

| RT (min) | m/z (Da) | Repeatability (RSD%) | | System stability (RSD%) | | Post-preparative stability (RSD%) | | Freeze-thaw stability (RSD%) | |
| --- | --- | --- | --- | --- | --- | --- | --- | --- | --- |
|  |  | RT | Area | RT | Area | RT | Area | RT | Area |
| 1.4975 | 642.18 | 1.92 | 6.95 | 0.49 | 3.28 | 0.82 | 9.34 | 0.59 | 8.66 |
| 3.6008 | 136.59 | 0.81 | 6.38 | 1.56 | 9.66 | 1.68 | 2.88 | 1.58 | 3.45 |
| 4.6961 | 241.66 | 0.37 | 7.26 | 0.96 | 5.34 | 0.86 | 3.95 | 2.08 | 7.62 |
| 6.3032 | 348.85 | 1.16 | 3.85 | 0.62 | 6.28 | 0.55 | 2.89 | 0.95 | 5.29 |
| 9.7821 | 276.50 | 0.66 | 8.69 | 1.09 | 2.98 | 1.35 | 6.17 | 1.34 | 2.18 |
| 11.5937 | 317.40 | 0.53 | 5.03 | 1.58 | 6.14 | 0.99 | 3.16 | 0.26 | 3.41 |

**Table S4** Parameters of the PCA and OPLS-DA models for NMR and UPLC-MS analysis

| Biological matrices | Detect | Model | R^2^X | R^2^Y | Q^2^ |
| --- | --- | --- | --- | --- | --- |
| Fecal | NMR | PCA | 0.668 | — | 0.432 |
|  |  | OPLS-DA (CON vs HF) | 0.767 | 0.998 | 0.856 |
|  |  | OPLS-DA (HF vs YCWLP) | 0.895 | 0.999 | 0.884 |
|  | MS | PCA | 0.708 | — | 0.386 |
|  |  | OPLS-DA (CON vs HF) | 0.807 | 0.999 | 0.883 |
|  |  | OPLS-DA (HF vs YCWLP) | 0.661 | 0.999 | 0.763 |

**Table S5** Key fecal metabolites with significant variations between the CON and HF groups

| No. | Metabolites | Detected | Retention time(min) /Chemical shift (ppm) | Measured mass (m/z) | VIP | Fold change (HF/CON) | *p*-value | HMDB |
| --- | --- | --- | --- | --- | --- | --- | --- | --- |
| 1 | Phenylacetic acid | MS | 3.60 | 137.05 | 1.44 | 1.55 | <0.05 | 0000209 |
| 2 | Pimelic acid | MS | 6.84 | 161.39 | 1.42 | 1.24 | <0.01 | 0000857 |
| 3 | 4,6-Dihydroxyquinoline | MS | 6.85 | 162.06 | 1.47 | 1.25 | <0.01 | 0004077 |
| 4 | 3-Methyldioxyindole | MS | 9.06 | 163.92 | 1.57 | 2.15 | <0.05 | 0004186 |
| 5 | Pantothenic acid | MS | 13.06 | 220.78 | 1.34 | 0.52 | <0.05 | 0000210 |
| 6 | Myristic acid | MS | 11.60 | 229.62 | 1.25 | 1.48 | <0.05 | 0000806 |
| 7 | Palmitoleic acid | MS | 16.16 | 255.76 | 1.37 | 1.85 | <0.01 | 0003229 |
| 8 | (R)-3-Hydroxy-hexadecanoic acid | MS | 11.56 | 273.24 | 1.38 | 1.75 | <0.01 | 0010734 |
| 9 | Alanyltryptophan | MS | 9.78 | 276.10 | 1.29 | 1.19 | <0.01 | 0013209 |
| 10 | Stearidonic acid | MS | 16.31 | 277.23 | 2.10 | 1.88 | <0.01 | 0006547 |
| 11 | α-Linolenic acid | MS | 15.92 | 279.23 | 1.33 | 1.62 | <0.05 | 0001388 |
| 12 | Linoleic acid | MS | 16.94 | 281.56 | 1.49 | 2.19 | <0.01 | 0000673 |
| 13 | Oleamide | MS | 16.94 | 282.29 | 1.48 | 1.82 | <0.01 | 0002117 |
| 14 | Sphingosine | MS | 16.96 | 300.31 | 1.50 | 2.83 | <0.01 | 0000252 |
| 15 | Sphinganine | MS | 12.03 | 302.30 | 1.36 | 1.32 | <0.05 | 0000269 |
| 16 | N-Acetylneuraminic acid | MS | 14.00 | 310.08 | 1.98 | 0.47 | <0.01 | 0000230 |
| 17 | Phytosphingosine | MS | 11.59 | 318.73 | 1.26 | 1.19 | <0.01 | 0004610 |
| 18 | Docosahexaenoic acid | MS | 12.79 | 329.57 | 1.42 | 1.65 | <0.01 | 0040611 |
| 19 | 14,15-DiHETrE | MS | 9.79 | 339.48 | 1.88 | 1.68 | <0.01 | 0002265 |
| 20 | Sucrose | MS | 9.78 | 343.33 | 1.40 | 1.45 | <0.05 | 0000258 |
| 21 | Adrenic acid | MS | 15.15 | 355.44 | 1.23 | 0.69 | <0.05 | 0002226 |
| 22 | Tetracosahexaenoic acid | MS | 13.83 | 357.63 | 1.64 | 1.42 | <0.01 | 0002007 |
| 23 | Rosmarinic acid | MS | 11.25 | 361.16 | 1.26 | 1.50 | <0.05 | 0003572 |
| 24 | 3-Oxo-4,6-choladienoic acid | MS | 14.25 | 371.33 | 1.32 | 0.73 | <0.05 | 0000476 |
| 25 | Cervonoyl ethanolamide | MS | 15.13 | 373.26 | 1.51 | 0.60 | <0.05 | 0013627 |
| 26 | MG(0:0/20:4(8Z,11Z,14Z,17Z)/0:0) | MS | 11.25 | 379.24 | 1.34 | 1.40 | <0.05 | 0011549 |
| 27 | Nutriacholic acid | MS | 15.12 | 413.56 | 1.89 | 0.40 | <0.05 | 0000467 |
| 28 | Chenodeoxycholic acid | MS | 15.01 | 415.42 | 1.22 | 0.67 | <0.05 | 0000518 |
| 29 | Cholic acid | MS | 12.00 | 430.85 | 2.39 | 0.34 | <0.01 | 0000619 |
| 30 | LysoPE(0:0/15:0) | MS | 14.42 | 440.76 | 1.96 | 2.63 | <0.01 | 0011472 |
| 31 | Coprocholic acid | MS | 9.78 | 452.33 | 1.29 | 1.61 | <0.05 | 0000601 |
| 32 | L-Urobilinogen | MS | 13.27 | 619.30 | 2.14 | 1.75 | <0.01 | 0004157 |
| 33 | Deoxycholate | NMR | 0.68 (s) | — | 1.35 | 0.06 | <0.05 | 0000626 |
| 34 | Butyrate | NMR | **0.92 (t),** 1.56 (m), 2.16 (t) | — | 1.71 | 0.63 | <0.01 | 0000039 |
| 35 | Citrulline | NMR | 1.57 (m) | — | 1.34 | 0.70 | <0.05 | 0000904 |
| 36 | Acetate | NMR | 1.92 (s) | — | 1.75 | 0.55 | <0.01 | 0000042 |
| 37 | Propionic acid | NMR | 1.06 (t), **2.19 (q)** | — | 1.50 | 0.74 | <0.05 | 0000237 |
| 38 | L-Glutamate | NMR | 2.08 (m), **2.36 (m)**, 3.75 (m) | — | 1.25 | 0.83 | <0.05 | 0060475 |
| 39 | Succinate | NMR | 2.41 (s) | — | 1.35 | 0.75 | <0.05 | 0000254 |
| 40 | L-Glutamine | NMR | 2.48 (m) | — | 1.53 | 0.49 | <0.01 | 0000641 |
| 41 | Dimethylamine (DMA) | NMR | 2.71 (s) | — | 1.30 | 0.69 | <0.05 | 0000087 |
| 42 | L-Aspartate | NMR | 2.80 (dd) | — | 1.56 | 0.72 | <0.01 | 0000191 |
| 43 | L-Tyrosine | NMR | 3.08 (m), **3.92 (m)**, 6.90 (d), 7.20(d) | — | 1.31 | 0.75 | <0.05 | 0000158 |
| 44 | Choline | NMR | 3.20 (s) | — | 1.70 | 1.92 | <0.01 | 0000097 |
| 45 | Glycine | NMR | 3.57 (s) | — | 1.45 | 0.68 | <0.05 | 0000123 |
| 46 | Glycerol | NMR | 3.68 (dd) | — | 1.34 | 0.88 | <0.05 | 0000131 |
| 47 | Creatine | NMR | 3.04 (s), **3.95 (s)** | — | 1.27 | 0.75 | <0.05 | 0000064 |
| 48 | Hippurate | NMR | 3.99 (d) | — | 1.27 | 0.70 | <0.05 | 0000714 |
| 49 | Creatinine | NMR | 4.08 (s) | — | 1.46 | 0.40 | <0.05 | 0000562 |
| 50 | L-Proline | NMR | 2.05 (m), 2.36 (m), 3.34 (m), **4.11 (m)** | — | 1.31 | 0.65 | <0.05 | 0000162 |
| 51 | L-Threonine | NMR | 1.33 (d), 3.59 (d), **4.26 (m)** | — | 1.53 | 0.59 | <0.05 | 0000167 |
| 52 | Glycerophosphocholine | NMR | 4.32 (dd) | — | 1.71 | 0.36 | <0.01 | 0000086 |
| 53 | β-Glucose | NMR | 4.66 (d) | — | 1.35 | 0.45 | <0.05 | 0000122 |
| 54 | Fumarate | NMR | 6.52 (s) | — | 1.80 | 2.69 | <0.01 | 0000134 |
| 55 | Hypoxanthine | NMR | **8.19 (s)**, 8.22 (s) | — | 1.52 | 1.71 | <0.05 | 0000157 |
| 56 | Adenosine | NMR | **8.27 (s)**, 8.38 (s) | — | 1.46 | 0.45 | <0.05 | 0000050 |
| 57 | Formate | NMR | 8.46 (s) | — | 1.24 | 32.33 | <0.01 | 0000142 |

The chemical shift values of bold type correspond to the VIP values in CON vs. HF group.

**Table S6** Key fecal metabolites with significant variations between the HF and YCWLP groups

| No. | Metabolites | Detected | Retention time(min) /Chemical shift (ppm) | Measured mass (m/z) | VIP | Fold change (YCWLP/HF) | *p*-value | HMDB |
| --- | --- | --- | --- | --- | --- | --- | --- | --- |
| 1 | Phenylacetic acid | MS | 3.60 | 137.05 | 1.34 | 0.59 | <0.05 | 0000209 |
| 2 | Guanine | MS | 2.83 | 152.55 | 1.43 | 0.61 | <0.05 | 0000132 |
| 3 | Pimelic acid | MS | 6.85 | 161.46 | 1.29 | 0.90 | <0.05 | 0000857 |
| 4 | 4,6-Dihydroxyquinoline | MS | 6.85 | 162.06 | 1.24 | 0.90 | <0.05 | 0004077 |
| 5 | 3-Methyldioxyindole | MS | 9.06 | 164.19 | 1.24 | 0.54 | <0.05 | 0004186 |
| 6 | Pantothenic acid | MS | 13.06 | 220.78 | 1.39 | 2.18 | <0.05 | 0000210 |
| 7 | Myristic acid | MS | 11.60 | 229.62 | 1.30 | 0.63 | <0.05 | 0000806 |
| 8 | Palmitoleic acid | MS | 16.16 | 255.76 | 1.97 | 0.55 | <0.01 | 0003229 |
| 9 | (R)-3-Hydroxy-hexadecanoic acid | MS | 11.56 | 273.24 | 1.82 | 0.54 | <0.01 | 0010734 |
| 10 | Alanyltryptophan | MS | 9.79 | 276.17 | 1.34 | 0.85 | <0.05 | 0013209 |
| 11 | Stearidonic acid | MS | 16.31 | 277.23 | 1.51 | 0.60 | <0.01 | 0006547 |
| 12 | Linoleic acid | MS | 16.94 | 281.56 | 1.87 | 0.49 | <0.01 | 0000673 |
| 13 | Oleamide | MS | 16.94 | 282.29 | 1.94 | 0.51 | <0.01 | 0002117 |
| 14 | Sphingosine | MS | 16.96 | 300.31 | 1.93 | 0.38 | <0.01 | 0000252 |
| 15 | Sphinganine | MS | 12.03 | 302.30 | 1.36 | 0.68 | <0.05 | 0000269 |
| 16 | N-Acetylneuraminic acid | MS | 14.00 | 310.08 | 1.32 | 1.98 | <0.05 | 0000230 |
| 17 | Phytosphingosine | MS | 11.59 | 318.80 | 1.36 | 0.85 | <0.05 | 0004610 |
| 18 | Docosahexaenoic acid | MS | 12.79 | 329.57 | 1.72 | 0.67 | <0.01 | 0040611 |
| 19 | Docosatrienoic acid | MS | 14.39 | 335.35 | 1.32 | 0.77 | <0.05 | 0002823 |
| 20 | 14,15-DiHETrE | MS | 9.79 | 339.48 | 1.39 | 0.62 | <0.05 | 0002265 |
| 21 | Sucrose | MS | 9.78 | 343.33 | 1.40 | 0.75 | <0.05 | 0000258 |
| 22 | Adrenic acid | MS | 15.14 | 355.57 | 1.21 | 1.74 | <0.05 | 0002226 |
| 23 | Tetracosahexaenoic acid | MS | 13.83 | 357.63 | 1.76 | 0.72 | <0.01 | 0002007 |
| 24 | Rosmarinic acid | MS | 11.25 | 361.09 | 1.36 | 0.61 | <0.05 | 0003572 |
| 25 | 3-Oxo-4,6-choladienoic acid | MS | 14.25 | 371.33 | 1.30 | 1.36 | <0.05 | 0000476 |
| 26 | Cervonoyl ethanolamide | MS | 15.13 | 373.26 | 1.23 | 1.71 | <0.05 | 0013627 |
| 27 | MG(0:0/20:4(8Z,11Z,14Z,17Z)/0:0) | MS | 11.25 | 379.31 | 1.43 | 0.58 | <0.05 | 0011549 |
| 28 | Nutriacholic acid | MS | 15.12 | 413.56 | 1.46 | 2.37 | <0.05 | 0000467 |
| 29 | Chenodeoxycholic acid | MS | 15.01 | 415.42 | 1.23 | 1.59 | <0.05 | 0000518 |
| 30 | Cholic acid | MS | 12.00 | 430.85 | 1.39 | 2.35 | <0.05 | 0000619 |
| 31 | LysoPE(0:0/15:0) | MS | 14.42 | 440.76 | 1.37 | 0.54 | <0.05 | 0011472 |
| 32 | LysoPE(0:0/18:1(11Z)) | MS | 11.23 | 480.59 | 1.82 | 0.57 | <0.01 | 0011475 |
| 33 | L-Urobilinogen | MS | 13.27 | 619.30 | 1.54 | 0.66 | <0.01 | 0004157 |
| 34 | 7-Ketolithocholic acid | MS | 16.43 | 781.50 | 1.50 | 0.46 | <0.01 | 0000467 |
| 35 | Deoxycholate | NMR | 0.68 (s) | — | 1.76 | 0.44 | <0.01 | 0000626 |
| 36 | Butyrate | NMR | **0.92 (t)**, 1.56 (m), 2.16 (t) | — | 1.79 | 2.08 | <0.01 | 0000039 |
| 37 | L-Alanine | NMR | 1.48 (d) | — | 1.59 | 1.28 | <0.05 | 0000161 |
| 38 | Citrulline | NMR | 1.57(m) | — | 1.71 | 1.55 | <0.01 | 0000904 |
| 39 | L-Lysine | NMR | **1.73 (m)** , 3.77 (t) | — | 1.62 | 0.74 | <0.05 | 0000182 |
| 40 | L-Glutamine | NMR | 2.48(m) | — | 1.39 | 1.53 | <0.01 | 0000641 |
| 41 | Choline | NMR | 3.20 (s) | — | 1.58 | 0.59 | <0.05 | 0000097 |
| 42 | L-Tyrosine | NMR | 3.08 (m), 3.92 (m), 6.90 (d), 7.20(d) | — | 2.08 | 1.20 | <0.05 | 0000158 |
| 43 | Creatine | NMR | 3.04 (s), 3.95 (s) | — | 1.65 | 1.21 | <0.01 | 0000064 |
| 44 | Creatinine | NMR | 4.08 (s) | — | 2.39 | 2.27 | <0.01 | 0000562 |
| 45 | L-Threonine | NMR | 1.33 (d), 3.59 (d), 4.26 (m) | — | 2.63 | 1.42 | <0.01 | 0000167 |
| 46 | Glycerophosphocholine | NMR | 4.32 (dd) | — | 1.43 | 2.07 | <0.05 | 0000086 |
| 47 | Fumarate | NMR | 6.52 (s) | — | 2.06 | 0.50 | <0.01 | 0000134 |
| 48 | L-Phenylalanine | NMR | 7.33 (m), 7.38 (m), 7.43 (m) | — | 1.60 | 0.80 | <0.05 | 0000159 |
| 49 | Hypoxanthine | NMR | **8.19 (s)**, 8.22 (s) | — | 1.45 | 0.42 | <0.01 | 0000157 |
| 50 | Formate | NMR | 8.46 (s) | — | 1.56 | 0.52 | <0.05 | 0000142 |

The chemical shift values of bold type correspond to the VIP values in HF vs. YCWLP group.

**Table S7** Key fecal metabolites significantly changed by CCl_4_ and reversed by YCWLP

| No. | Metabolites | Detected | Retention time(min) /Chemical shift (ppm) | Measured mass (m/z) | Fold change (Trend) | |
| --- | --- | --- | --- | --- | --- | --- |
|  |  |  |  |  | HF/CON | YCWLP/HF |
| 1 | Phenylacetic acid | MS | 3.60 | 137.05 | 1.55 (↑^*^) | 0.59 (↓^#^) |
| 2 | Pimelic acid | MS | 6.85 | 161.46 | 1.24 (↑^**^) | 0.90 (↓^#^) |
| 3 | 4,6-Dihydroxyquinoline | MS | 6.85 | 162.06 | 1.25 (↑^**^) | 0.90 (↓^#^) |
| 4 | 3-Methyldioxyindole | MS | 9.06 | 164.19 | 2.15 (↑^*^) | 0.54 (↓^#^) |
| 5 | Pantothenic acid | MS | 13.06 | 220.78 | 0.52 (↓^*^) | 2.18 (↑^#^) |
| 6 | Myristic acid | MS | 11.60 | 229.62 | 1.48 (↑^*^) | 0.63 (↓^#^) |
| 7 | Palmitoleic acid | MS | 16.16 | 255.76 | 1.85 (↑^**^) | 0.55 (↓^##^) |
| 8 | (R)-3-Hydroxy-hexadecanoic acid | MS | 11.56 | 273.24 | 1.75 (↑^**^) | 0.54 (↓^##^) |
| 9 | Alanyltryptophan | MS | 9.79 | 276.17 | 1.19 (↑^**^) | 0.85 (↓^#^) |
| 10 | Stearidonic acid | MS | 16.31 | 277.23 | 1.88 (↑^**^) | 0.60 (↓^##^) |
| 11 | Linoleic acid | MS | 16.94 | 281.56 | 2.19 (↑^**^) | 0.49 (↓^##^) |
| 12 | Oleamide | MS | 16.94 | 282.29 | 1.82 (↑^**^) | 0.51 (↓^##^) |
| 13 | Sphingosine | MS | 16.96 | 300.31 | 2.83 (↑^**^) | 0.38 (↓^##^) |
| 14 | Sphinganine | MS | 12.03 | 302.30 | 1.32 (↑^*^) | 0.68 (↓^#^) |
| 15 | N-Acetylneuraminic acid | MS | 14.00 | 310.08 | 0.47 (↓^**^) | 1.98 (↑^#^) |
| 16 | Phytosphingosine | MS | 11.59 | 318.80 | 1.19 (↑^**^) | 0.85 (↓^#^) |
| 17 | Docosahexaenoic acid | MS | 12.79 | 329.57 | 1.65 (↑^**^) | 0.67 (↓^##^) |
| 18 | 14,15-DiHETrE | MS | 9.79 | 339.48 | 1.68 (↑^**^) | 0.62 (↓^#^) |
| 19 | Sucrose | MS | 9.78 | 343.33 | 1.45 (↑^*^) | 0.75 (↓^#^) |
| 20 | Adrenic acid | MS | 15.14 | 355.57 | 0.69 (↓^*^) | 1.74 (↑^#^) |
| 21 | Tetracosahexaenoic acid | MS | 13.83 | 357.63 | 1.42 (↑^**^) | 0.72 (↓^##^) |
| 22 | Rosmarinic acid | MS | 11.25 | 361.09 | 1.50 (↑^*^) | 0.61 (↓^#^) |
| 23 | 3-Oxo-4,6-choladienoic acid | MS | 14.25 | 371.33 | 0.73 (↓^*^) | 1.36 (↑^#^) |
| 24 | Cervonoyl ethanolamide | MS | 15.13 | 373.26 | 0.60 (↓^*^) | 1.71 (↑^#^) |
| 25 | MG(0:0/20:4(8Z,11Z,14Z,17Z)/0:0) | MS | 11.25 | 379.31 | 1.40 (↑^*^) | 0.58 (↓^#^) |
| 26 | Nutriacholic acid | MS | 15.12 | 413.56 | 0.40 (↓^*^) | 2.37 (↑^#^) |
| 27 | Chenodeoxycholic acid | MS | 15.01 | 415.42 | 0.67 (↓^*^) | 1.59 (↑^#^) |
| 28 | Cholic acid | MS | 12.00 | 430.85 | 0.34 (↓^**^) | 2.35 (↑^#^) |
| 29 | LysoPE(0:0/15:0) | MS | 14.42 | 440.76 | 2.63 (↑^**^) | 0.54 (↓^#^) |
| 30 | L-Urobilinogen | MS | 13.27 | 619.30 | 1.75 (↑^**^) | 0.66 (↓^##^) |
| 31 | Deoxycholate | NMR | 0.68 (s) | — | 0.66 (↓^*^) | 1.44 (↑^##^) |
| 32 | Butyrate | NMR | **0.92 (t)**, 1.56 (m), 2.16 (t) | — | 0.63 (↓^**^) | 2.08 (↑^##^) |
| 33 | Citrulline | NMR | 1.57(m) | — | 0.70 (↓^*^) | 1.55 (↑^##^) |
| 34 | L-Glutamine | NMR | 2.48(m) | — | 0.49 (↓^**^) | 1.53 (↑^##^) |
| 35 | L-Tyrosine | NMR | 3.08 (m), **3.92 (m)**, 6.90 (d), 7.20(d) | — | 0.75 (↓^*^) | 1.20 (↑^#^) |
| 36 | Creatine | NMR | 3.04 (s), **3.95 (s)** | — | 0.75 (↓^*^) | 1.21 (↑^##^) |
| 37 | Creatinine | NMR | 4.08 (s) | — | 0.40 (↓^*^) | 2.27 (↑^##^) |
| 38 | L-Threonine | NMR | 1.33 (d), 3.59 (d), **4.26 (m)** | — | 0.59 (↓^*^) | 1.42 (↑^##^) |
| 39 | Glycerophosphocholine | NMR | 4.32 (dd) | — | 0.36 (↓^**^) | 2.07 (↑^#^) |
| 40 | Fumarate | NMR | 6.52 (s) | — | 2.69 (↑^**^) | 0.50 (↓^##^) |
| 41 | Hypoxanthine | NMR | **8.19 (s)**, 8.22 (s) | — | 1.71 (↑^*^) | 0.42 (↓^##^) |
| 42 | Formate | NMR | 8.46 (s) | — | 32.33 (↑^**^) | 0.52 (↓^#^) |

The peak area corresponding to the chemical shift value in bold was used to calculate the fold change between different groups. “↑” and “↓” means the metabolite is up-regulated and down-regulated. Compared with the CON group: ^*^, *p*-value < 0.05; ^**^, *p*-value < 0.01. Compared with the HF group: ^#^, *p*-value < 0.05; ^##^, *p*-value < 0.01.

**Table S8** Alpha diversity analysis of microbial community structure of the CON, HF and YCWLP groups

|  | Chao1 | Shannon | Simpson | PD_whole_tree |
| --- | --- | --- | --- | --- |
| CON | 10802.48 ± 674.03^**^ | 9.31 ± 0.40^**^ | 0.99 ± 0.0052 | 254.24 ± 4.19 |
| HF | 8687.68 ± 1080.60 | 8.59 ± 0.23 | 0.98 ± 0.0075 | 246.83 ± 18.67 |
| YCWLP | 8711.86 ± 908.29 | 9.11 ± 0.32^*^ | 0.98 ± 0.0057 | 249.01 ± 12.74 |

Significant difference compared with the HF group: ^*^ *p*-value < 0.05; ^**^ *p*-value < 0.01.

**Table S9** Spearman’s correlation coefficients of pathological indices, key fecal metabolites and significantly changed genera.

|  | Index | Rothia | Prevotella | Streptococcus | | Robinsoniella | | rc4-4 | | Butyricicoccus | | Faecalibacterium | | Christensenella | | Anaerostipes | | Coprococcus | | Bifidobacterium | | Oscillospira | | Allobaculum | | Bilophila | | Desulfovibrio | | Barnesiella | | Dehalobacterium | | Roseburia | | [Ruminococcus] |
| --- | --- | --- | --- | --- | --- | --- | --- | --- | --- | --- | --- | --- | --- | --- | --- | --- | --- | --- | --- | --- | --- | --- | --- | --- | --- | --- | --- | --- | --- | --- | --- | --- | --- | --- | --- | --- |
| Pharmacodynamic parameters | Liver Index (%) | -0.7100 | -0.2750 | -0.1950 | | -0.4960 | | -0.4500 | | -0.4180 | | -0.4820 | | -0.1250 | | -0.2610 | | -0.2140 | | 0.0660 | | 0.5930 | | 0.0370 | | 0.4620 | | 0.4820 | | 0.5320 | | 0.2930 | | 0.1890 | | 0.5320 |
|  | Spleen Index (%) | -0.6090 | -0.2360 | -0.2200 | | -0.6930 | | -0.7000 | | -0.4390 | | -0.7610 | | -0.6450 | | 0.0540 | | -0.3000 | | 0.1000 | | 0.3890 | | 0.3020 | | 0.7460 | | 0.6680 | | 0.2210 | | 0.5360 | | 0.7250 | | 0.3680 |
|  | Thymus Index (%) | 0.6120 | 0.5100 | -0.0170 | | 0.7650 | | 0.5420 | | 0.5010 | | 0.4170 | | 0.5170 | | 0.1880 | | 0.2840 | | 0.0430 | | -0.4240 | | -0.2480 | | -0.5920 | | -0.6300 | | -0.2430 | | -0.5620 | | -0.4280 | | -0.4060 |
|  | ALT(U/L) | -0.5630 | -0.4180 | -0.3980 | | -0.7490 | | -0.6640 | | -0.6390 | | -0.6820 | | -0.6120 | | -0.1360 | | -0.2890 | | 0.0870 | | 0.5960 | | 0.4280 | | 0.6880 | | 0.7320 | | 0.3570 | | 0.4000 | | 0.5610 | | 0.5890 |
|  | AST(U/L) | -0.5340 | -0.3860 | -0.4160 | | -0.6890 | | -0.7180 | | -0.6140 | | -0.6210 | | -0.6250 | | -0.1290 | | -0.4430 | | -0.0100 | | 0.5500 | | 0.3720 | | 0.6160 | | 0.5890 | | 0.3890 | | 0.4460 | | 0.5750 | | 0.5240 |
|  | HA(ng/L) | -0.5770 | -0.5750 | -0.2070 | | -0.6040 | | -0.4960 | | -0.6460 | | -0.5000 | | -0.3750 | | -0.0960 | | -0.3250 | | 0.0490 | | 0.5640 | | 0.3310 | | 0.6850 | | 0.7000 | | 0.3110 | | 0.5210 | | 0.4500 | | 0.4500 |
|  | LN(ng/L) | -0.6540 | -0.3790 | -0.4520 | | -0.7290 | | -0.7430 | | -0.3960 | | -0.7930 | | -0.5560 | | -0.1140 | | -0.3930 | | 0.0330 | | 0.5210 | | 0.3680 | | 0.6380 | | 0.6460 | | 0.5360 | | 0.3680 | | 0.6360 | | 0.4250 |
|  | CIV(μg/L) | -0.5740 | -0.4750 | -0.3710 | | -0.6080 | | -0.5710 | | -0.5680 | | -0.7110 | | -0.4920 | | 0.0320 | | -0.3610 | | 0.0430 | | 0.5750 | | 0.4730 | | 0.7990 | | 0.7210 | | 0.3570 | | 0.5290 | | 0.6750 | | 0.4750 |
|  | PⅢNP(μg/L) | -0.5810 | -0.5570 | -0.2230 | | -0.8100 | | -0.6680 | | -0.4250 | | -0.7000 | | -0.5200 | | -0.0140 | | -0.3000 | | 0.0820 | | 0.4540 | | 0.3890 | | 0.6630 | | 0.7430 | | 0.2710 | | 0.5500 | | 0.6110 | | 0.3860 |
|  | α-SMA(pg/mL) | -0.5660 | -0.4600 | -0.2490 | | -0.6680 | | -0.6190 | | -0.5690 | | -0.7570 | | -0.5650 | | 0.0470 | | -0.2880 | | 0.0700 | | 0.4540 | | 0.3640 | | 0.7830 | | 0.7670 | | 0.2810 | | 0.5330 | | 0.6820 | | 0.4080 |
|  | TIMP-1(ng/mL) | -0.5420 | -0.5580 | -0.3730 | | -0.6570 | | -0.5450 | | -0.6330 | | -0.6670 | | -0.5440 | | -0.0750 | | -0.3180 | | 0.0430 | | 0.6560 | | 0.4490 | | 0.6670 | | 0.7080 | | 0.3990 | | 0.4540 | | 0.5740 | | 0.5810 |
|  | PDGF(pg/mL) | -0.6800 | -0.4720 | -0.1700 | | -0.4210 | | -0.3560 | | -0.6170 | | -0.4080 | | -0.3150 | | -0.2310 | | -0.3430 | | -0.1510 | | 0.4950 | | 0.2020 | | 0.7050 | | 0.5760 | | 0.5660 | | 0.3500 | | 0.3090 | | 0.5400 |
|  | TGF-β1(ng/mL) | -0.5830 | -0.4750 | -0.2020 | | -0.7780 | | -0.6070 | | -0.6070 | | -0.5430 | | -0.5680 | | -0.0820 | | -0.2960 | | 0.1050 | | 0.6210 | | 0.4220 | | 0.6770 | | 0.6710 | | 0.2570 | | 0.5750 | | 0.4960 | | 0.5570 |
|  | LPS(pg/mL) | -0.6310 | -0.4430 | -0.0320 | | -0.5040 | | -0.4460 | | -0.3820 | | -0.2000 | | -0.3510 | | -0.5460 | | -0.4750 | | -0.3910 | | 0.2000 | | -0.1220 | | 0.3050 | | 0.3820 | | 0.6110 | | 0.2540 | | 0.1110 | | 0.4390 |
| Key metabolites | Phenylacetic acid | -0.2970 | -0.3540 | -0.2650 | | -0.0770 | | -0.1500 | | -0.1820 | | 0.1460 | | 0.1690 | | -0.4250 | | -0.4360 | | -0.2320 | | 0.1890 | | 0.5800 | | 0.0860 | | 0.1000 | | 0.4290 | | -0.0500 | | -0.1610 | | 0.2000 |
|  | Pimelic acid | -0.3520 | -0.5610 | -0.4590 | | -0.6080 | | -0.6610 | | -0.2570 | | -0.5070 | | -0.2340 | | 0.0820 | | -0.4320 | | 0.1560 | | 0.2640 | | 0.4010 | | 0.4840 | | 0.5570 | | 0.2180 | | 0.4070 | | 0.3860 | | 0.3540 |
|  | 4,6-Dihydroxyquinoline | -0.3990 | -0.5860 | -0.4880 | | -0.6650 | | -0.6930 | | -0.3140 | | -0.5750 | | -0.2900 | | 0.0540 | | -0.4210 | | 0.1560 | | 0.3360 | | 0.4440 | | 0.5380 | | 0.6140 | | 0.2540 | | 0.4040 | | 0.4250 | | 0.3960 |
|  | 3-Methyldioxyindole | -0.6430 | -0.1790 | -0.3600 | | -0.3340 | | -0.5040 | | -0.3680 | | -0.4320 | | -0.0970 | | -0.3290 | | -0.3680 | | -0.0510 | | 0.3320 | | -0.0760 | | 0.4160 | | 0.4180 | | 0.5180 | | 0.1140 | | 0.1680 | | 0.3960 |
|  | Pantothenic acid | 0.4840 | 0.1460 | -0.0380 | | 0.2300 | | 0.3110 | | 0.4140 | | -0.0390 | | -0.0320 | | 0.3390 | | 0.4110 | | 0.1230 | | -0.2680 | | -0.0600 | | -0.4980 | | -0.2640 | | -0.3570 | | -0.2710 | | 0.1110 | | -0.7180 |
|  | Myristic acid | -0.4630 | 0.0460 | -0.1910 | | -0.0040 | | -0.4570 | | -0.0610 | | -0.0890 | | 0.1410 | | -0.2890 | | -0.5930 | | -0.1230 | | -0.1360 | | -0.2230 | | 0.3260 | | 0.1320 | | 0.3820 | | 0.0500 | | -0.0250 | | 0.3320 |
|  | Palmitoleic acid | -0.6720 | -0.3640 | -0.0540 | | -0.4230 | | -0.4110 | | -0.4460 | | -0.2890 | | -0.2500 | | -0.3540 | | -0.3930 | | -0.1660 | | 0.3860 | | 0.0930 | | 0.5770 | | 0.4250 | | 0.4610 | | 0.2750 | | 0.1140 | | 0.6640 |
|  | (R)-3-Hydroxy-hexadecanoic acid | -0.6010 | 0.0820 | -0.0670 | | -0.1330 | | -0.5860 | | -0.3180 | | -0.2710 | | -0.2380 | | -0.5210 | | -0.7000 | | -0.3880 | | 0.0320 | | -0.3270 | | 0.3940 | | 0.1680 | | 0.6930 | | 0.1390 | | 0.1110 | | 0.6750 |
|  | Alanyltryptophan | -0.4590 | -0.5290 | -0.5080 | | -0.7130 | | -0.7250 | | -0.2460 | | -0.5460 | | -0.3670 | | -0.1110 | | -0.3890 | | 0.0610 | | 0.2290 | | 0.3820 | | 0.4910 | | 0.5680 | | 0.2820 | | 0.2070 | | 0.3360 | | 0.3500 |
|  | Stearidonic acid | -0.7690 | -0.1290 | -0.3030 | | -0.3420 | | -0.5930 | | -0.4500 | | -0.2140 | | -0.3910 | | -0.6390 | | -0.6360 | | -0.3190 | | 0.2070 | | -0.0600 | | 0.5160 | | 0.3180 | | 0.5890 | | 0.0070 | | 0.1250 | | 0.4570 |
|  | Linoleic acid | -0.4090 | -0.3430 | -0.1950 | | -0.3910 | | -0.5210 | | -0.5890 | | -0.2820 | | -0.3420 | | -0.2750 | | -0.5890 | | -0.3040 | | 0.2960 | | 0.1590 | | 0.5340 | | 0.4110 | | 0.6000 | | 0.4040 | | 0.2500 | | 0.7570 |
|  | Oleamide | -0.4310 | -0.3320 | -0.1660 | | -0.3830 | | -0.5320 | | -0.4860 | | -0.2570 | | -0.3260 | | -0.2960 | | -0.5820 | | -0.4620 | | 0.2110 | | 0.0080 | | 0.3840 | | 0.2140 | | 0.6070 | | 0.3360 | | 0.1820 | | 0.7180 |
|  | Sphingosine | -0.7130 | 0.0430 | 0.1870 | | -0.4110 | | -0.6460 | | -0.4570 | | -0.4070 | | -0.6810 | | -0.4640 | | -0.5610 | | -0.3730 | | 0.1390 | | -0.0170 | | 0.6270 | | 0.3430 | | 0.5360 | | 0.1460 | | 0.4070 | | 0.5750 |
|  | Sphinganine | -0.4580 | -0.1180 | 0.0720 | | -0.2380 | | -0.3250 | | -0.4070 | | -0.1500 | | -0.0600 | | -0.1960 | | -0.2390 | | -0.0310 | | 0.1460 | | -0.0520 | | 0.5270 | | 0.3320 | | 0.2360 | | 0.2390 | | -0.0540 | | 0.6500 |
|  | N-Acetylneuraminic acid | 0.3200 | 0.3710 | -0.2380 | | 0.2180 | | 0.2320 | | 0.4820 | | 0.2960 | | 0.0320 | | 0.0860 | | 0.2040 | | 0.0840 | | -0.1610 | | 0.1080 | | -0.4770 | | -0.4890 | | -0.3140 | | -0.4430 | | -0.1880 | | -0.5750 |
|  | Phytosphingosine | -0.5740 | -0.3290 | -0.0580 | | -0.5920 | | -0.5110 | | -0.3930 | | -0.3140 | | -0.2100 | | 0.2000 | | -0.2000 | | 0.1940 | | 0.3000 | | 0.1880 | | 0.6160 | | 0.5890 | | 0.1430 | | 0.3070 | | 0.0750 | | 0.5680 |
|  | Docosahexaenoic acid | -0.6540 | -0.2500 | -0.2580 | | -0.2780 | | -0.3610 | | -0.6180 | | -0.5680 | | -0.3870 | | 0.2180 | | -0.3540 | | 0.1250 | | 0.5890 | | 0.1410 | | 0.6560 | | 0.5110 | | 0.5460 | | 0.2790 | | 0.4610 | | 0.4890 |
|  | 14,15-DiHETrE | -0.5770 | -0.3820 | -0.2140 | | -0.3140 | | -0.4960 | | -0.2610 | | -0.4390 | | -0.0850 | | -0.1610 | | -0.4820 | | -0.2370 | | 0.1320 | | -0.0790 | | 0.4300 | | 0.3790 | | 0.4790 | | 0.2930 | | 0.3140 | | 0.2860 |
|  | Sucrose | -0.3400 | -0.4320 | -0.3590 | | -0.2220 | | -0.4430 | | -0.0110 | | -0.3180 | | 0.1290 | | 0.0040 | | -0.4210 | | -0.1510 | | -0.0110 | | -0.0600 | | 0.1540 | | 0.1500 | | 0.2890 | | 0.1460 | | 0.1460 | | 0.1040 |
| Key metabolites | Adrenic acid | 0.0320 | 0.2320 | -0.3600 | -0.0640 | | -0.0180 | | 0.1790 | | 0.1710 | | -0.3020 | | -0.2070 | | -0.0570 | | -0.0690 | | 0.1070 | | 0.2360 | | -0.2470 | | -0.2820 | | 0.0180 | | -0.3290 | | 0.0110 | | -0.2390 | |
|  | Tetracosahexaenoic acid | -0.4330 | -0.4570 | -0.2140 | -0.6000 | | -0.6140 | | -0.3460 | | -0.6140 | | -0.6570 | | -0.2930 | | -0.4540 | | -0.2830 | | 0.2680 | | 0.0950 | | 0.3050 | | 0.4570 | | 0.5570 | | 0.2960 | | 0.5140 | | 0.3500 | |
|  | Rosmarinic acid | -0.5080 | -0.1680 | -0.0410 | -0.2010 | | -0.4320 | | -0.0610 | | -0.0290 | | -0.1410 | | -0.5110 | | -0.5680 | | -0.3240 | | -0.1680 | | -0.3740 | | 0.0970 | | 0.1140 | | 0.4710 | | 0.0570 | | -0.0610 | | 0.2250 | |
|  | 3-Oxo-4,6-choladienoic acid | 0.2520 | 0.0360 | -0.0670 | 0.0480 | | 0.3960 | | -0.0890 | | 0.3290 | | 0.2300 | | 0.0930 | | 0.3040 | | 0.2320 | | 0.3570 | | 0.4610 | | -0.0040 | | 0.0140 | | -0.0860 | | -0.0250 | | -0.2540 | | 0.2000 | |
|  | Cervonoyl ethanolamide | 0.3430 | 0.3930 | -0.1590 | 0.1850 | | 0.1820 | | 0.4320 | | 0.2930 | | -0.0890 | | 0.0570 | | 0.1460 | | 0.0310 | | -0.1930 | | 0.0600 | | -0.4870 | | -0.4860 | | -0.2860 | | -0.3610 | | -0.0640 | | -0.5320 | |
|  | MG(0:0/20:4(8Z,11Z,14Z,17Z)/0:0) | -0.5330 | -0.1250 | 0.0200 | -0.2580 | | -0.5070 | | -0.1000 | | 0.0710 | | -0.1850 | | -0.6820 | | -0.6320 | | -0.4340 | | -0.1960 | | -0.4420 | | 0.0570 | | 0.0290 | | 0.5460 | | 0.0070 | | -0.2210 | | 0.4390 | |
|  | Nutriacholic acid | 0.3810 | 0.1860 | 0.0380 | 0.1090 | | 0.3210 | | 0.5710 | | 0.3540 | | 0.0360 | | 0.0430 | | 0.3000 | | 0.0360 | | -0.2250 | | 0.0230 | | -0.6270 | | -0.4460 | | -0.3360 | | -0.3360 | | -0.1680 | | -0.6360 | |
|  | Chenodeoxycholic acid | 0.3220 | 0.3540 | -0.3080 | 0.0640 | | 0.0040 | | 0.4860 | | 0.0860 | | -0.0930 | | 0.1070 | | 0.1750 | | 0.1170 | | -0.2360 | | 0.0250 | | -0.5090 | | -0.4180 | | -0.3000 | | -0.4140 | | -0.0180 | | -0.5680 | |
|  | Cholic acid | 0.5740 | 0.5070 | 0.0830 | 0.3910 | | 0.2790 | | 0.5430 | | 0.3820 | | 0.1130 | | 0.1820 | | 0.2180 | | -0.1400 | | -0.5430 | | -0.2940 | | -0.7030 | | -0.6960 | | -0.3070 | | -0.3360 | | -0.2110 | | -0.5140 | |
|  | LysoPE(0:0/15:0) | -0.4840 | -0.3360 | -0.4630 | -0.3670 | | -0.5110 | | -0.3140 | | -0.6180 | | -0.2940 | | -0.2140 | | -0.4930 | | -0.1710 | | 0.4110 | | 0.1700 | | 0.4090 | | 0.4430 | | 0.7140 | | 0.2070 | | 0.4040 | | 0.5290 | |
|  | L-Urobilinogen | -0.5170 | -0.6390 | -0.4250 | -0.5880 | | -0.5960 | | -0.2460 | | -0.4790 | | -0.1130 | | -0.0860 | | -0.4430 | | 0.0130 | | 0.3290 | | 0.2960 | | 0.4620 | | 0.5290 | | 0.3820 | | 0.3250 | | 0.2710 | | 0.4070 | |
|  | Deoxycholate | 0.6560 | 0.3250 | 0.3670 | 0.6350 | | 0.6770 | | 0.3320 | | 0.6950 | | 0.4820 | | -0.0110 | | 0.4150 | | 0.1160 | | -0.3700 | | -0.2760 | | -0.6290 | | -0.5160 | | -0.3650 | | -0.4600 | | -0.7030 | | -0.2550 | |
|  | Butyrate | 0.5950 | 0.3860 | 0.4070 | 0.5600 | | 0.6710 | | 0.3000 | | 0.6290 | | 0.2340 | | 0.5820 | | 0.5960 | | 0.2250 | | -0.4290 | | -0.1100 | | -0.3980 | | -0.3390 | | -0.5930 | | -0.4210 | | -0.4390 | | -0.5460 | |
|  | Citrulline | 0.5770 | 0.3610 | 0.3350 | 0.6610 | | 0.7210 | | 0.2210 | | 0.6960 | | 0.2300 | | -0.0540 | | 0.5610 | | -0.0280 | | -0.4000 | | -0.1610 | | -0.4440 | | -0.4430 | | -0.3500 | | -0.4860 | | -0.4820 | | -0.5430 | |
|  | L-Glutamine | 0.7100 | 0.3180 | 0.2960 | 0.6290 | | 0.6610 | | 0.4430 | | 0.6210 | | 0.5640 | | 0.0290 | | 0.3610 | | 0.0820 | | -0.3610 | | -0.1700 | | -0.6380 | | -0.4860 | | -0.2430 | | -0.4360 | | -0.6110 | | -0.2390 | |
|  | L-Tyrosine | 0.2840 | 0.5140 | 0.1620 | 0.8100 | | 0.6210 | | 0.0250 | | 0.2640 | | 0.3510 | | 0.0210 | | 0.3570 | | 0.0740 | | -0.3290 | | -0.2540 | | -0.0070 | | -0.1180 | | -0.1890 | | -0.5180 | | -0.2110 | | -0.4390 | |
|  | Creatine | 0.6310 | 0.1930 | 0.1730 | 0.8180 | | 0.8070 | | 0.1930 | | 0.5040 | | 0.5600 | | 0.2390 | | 0.3500 | | -0.0230 | | -0.2430 | | -0.1160 | | -0.4160 | | -0.4070 | | -0.2390 | | -0.3360 | | -0.3210 | | -0.4710 | |
|  | Creatinine | 0.5900 | 0.3460 | -0.0410 | 0.7490 | | 0.5890 | | 0.1960 | | 0.2610 | | 0.4790 | | 0.2710 | | 0.2790 | | 0.2550 | | -0.2390 | | 0.0540 | | -0.1760 | | -0.1360 | | -0.2210 | | -0.3680 | | -0.1540 | | -0.4110 | |
|  | L-Threonine | 0.7080 | 0.1820 | 0.1660 | 0.7410 | | 0.6890 | | 0.2320 | | 0.4390 | | 0.5360 | | 0.3000 | | 0.2460 | | 0.0610 | | -0.1610 | | 0.0190 | | -0.4090 | | -0.3930 | | -0.2210 | | -0.2070 | | -0.2570 | | -0.2930 | |
|  | Glycerophosphocholine | 0.5630 | 0.3790 | -0.2200 | 0.7490 | | 0.4890 | | 0.1860 | | 0.3040 | | 0.3830 | | 0.1430 | | 0.2960 | | 0.1120 | | -0.3070 | | -0.0640 | | -0.3300 | | -0.4140 | | -0.2860 | | -0.6960 | | -0.3540 | | -0.4210 | |
|  | Fumarate | -0.6420 | -0.2040 | 0.0050 | -0.4510 | | -0.5320 | | -0.5070 | | -0.3430 | | -0.4590 | | -0.3710 | | -0.4430 | | -0.2090 | | 0.1360 | | -0.0520 | | 0.6160 | | 0.5540 | | 0.4210 | | 0.3500 | | 0.3540 | | 0.3750 | |
|  | Hypoxanthine | -0.2590 | 0.0430 | 0.3420 | -0.3140 | | -0.3250 | | -0.3000 | | -0.0930 | | -0.6450 | | -0.4070 | | -0.1640 | | -0.0870 | | -0.2290 | | -0.1430 | | 0.3440 | | 0.4000 | | 0.0210 | | 0.1000 | | 0.1960 | | 0.0460 | |
|  | Formate | -0.6450 | -0.3010 | -0.0730 | -0.4260 | | -0.3040 | | -0.5420 | | -0.3790 | | 0.3710 | | -0.2740 | | -0.1880 | | -0.0860 | | 0.3290 | | 0.0270 | | 0.6010 | | 0.6170 | | 0.4030 | | 0.2900 | | 0.2840 | | 0.3490 | |

**Figure S1** Representative ^1^H NMR spectra **(A)** and total ion chromatograms **(B)** of the fecal samples of the CON, HF and YCWLP groups. The number in the figure above represents the metabolite identified based on the ^1^H NMR spectra, and the detailed information is described in Table S2.

**Figure S2** OPLS-DA loading plots and PLS-DA permutation test plots (999 permutations) based on ^1^H NMR and UPLC-MS. OPLS-DA loading plots **(A)** and PLS-DA permutation test plots **(E)** of the CON and HF groups based on ^1^H NMR; OPLS-DA loading plots **(B)** and PLS-DA permutation test plots **(F)** of the HF and YCWLP groups based on ^1^H NMR; OPLS-DA loading plots **(C)** and PLS-DA permutation test plots **(G)** of the CON and HF groups based on UPLC-MS; OPLS-DA loading plots **(D)** and PLS-DA permutation test plots **(H)** of the HF and YCWLP groups based on UPLC-MS.

**Figure S3** The rarefaction curves of 16S rRNA gene sequencing analysis.

**Figure S4** Changes of the gut microbiota profile among the CON, HF and YCWLP groups based on 16S rRNA gene sequencing. **(A)** Alpha diversity analysis; **(B)** Unweighted UniFrac-based-PCoA; **(C)** Hierarchical clustering analysis of UPGMA; **(D)** NMDS analysis (stress=0.126); **(E)** ANOSIM analysis (R=0.639, P=0.001). Significant difference compared with the HF group: ^*^ *p*-value < 0.05; ^**^ *p*-value < 0.01.

**Figure S5** Differences in gut microbiota composition among the CON, HF and YCWLP groups. **(A)** Top ten species in the relative abundance at the phylum, class, order and family levels; **(B)** Species with significant changes among the experimental groups at the phylum, class, order and family levels. Significant difference compared with the HF group: ^*^ *p*-value < 0.05; ^**^ *p*-value < 0.01.

**Figure S5** Continued

**Figure S6** The relative abundance of genera with significant variations among the CON, HF and YCWLP groups. Significant difference compared with the HF group: ^*^ *p*-value < 0.05; ^**^ *p*-value < 0.01.
